# Asparaginase and Autophagy Inhibitors Effectively Remove Senescent Cells by Synergistically Limiting Asparagine Supply

**DOI:** 10.1111/acel.70203

**Published:** 2025-09-04

**Authors:** Zhihua Huang, Xinxin Liu, Xiaojia Zhou, Keyu Chen, Honglin Diao, Mingyue Wang, Jianlei Wei, Zeping Li, Yang Yang, Zebin Mao, Wenhua Yu

**Affiliations:** ^1^ Department of Biochemistry and Molecular Biology, School of Basic Medical Sciences Peking University Beijing China; ^2^ Department of General Surgery Peking University First Hospital Beijing China; ^3^ Department of Immunology, School of Basic Medicine Jiamusi University Jiamusi Heilongjiang Province China

**Keywords:** asparaginase, asparagine, asparagine synthetase, autophagy inhibitors, senescent cell

## Abstract

The accumulation of senescent cells (SNCs) contributes to tissue dysfunction and age‐related diseases, creating an urgent need for effective senolytic strategies. We identified a metabolic vulnerability in SNCs characterized by marked downregulation of asparagine synthetase (ASNS), rendering them uniquely dependent on exogenous asparagine (Asn). This vulnerability was exploited through combined treatment with L‐asparaginase (ASNase) and autophagy inhibitors, which synergistically deplete Asn via complementary mechanisms: ASNase degrades extracellular Asn pools, while autophagy inhibition blocks intracellular protein recycling as an alternative Asn source. This dual approach induced selective synthetic lethality across multiple SNC types in vitro. In aged mice, the combination therapy significantly reduced SNC burden in diverse tissues, improved physiological function, and attenuated progression of age‐related conditions including osteoporosis, atherosclerosis, and non‐alcoholic fatty liver disease. Our findings establish concurrent targeting of extracellular and intracellular Asn supplies as a potent, selective senolytic strategy with broad therapeutic potential for age‐related disorders.

## Graphical abstract

A model of cellular asparagine source and AH treatment kills senescent cells effectively and selectively. In senescent cells, there is a defect in ASNS. Treatment with ASNase to deplete extracellular Asn, combined with HCQ to inhibit autophagy‐derived Asn, results in a severe depletion of Asn and subsequent senescent cell death.

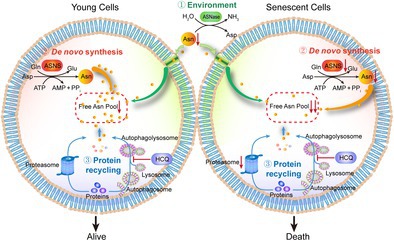

## 1 Introduction

Cellular senescence, characterized by irreversible cell cycle arrest in response to stress and damage, represents a double‐edged sword in organismal physiology (Childs et al. 2017). While transient senescence aids tumor suppression and wound healing, persistent senescent cells (SNCs) accumulate with age through diminished immune clearance (Robbins et al. 2021) and propagate detrimental effects via the senescence‐associated secretory phenotype (SASP) (Lucas et al. 2023). This accumulation drives tissue dysfunction and accelerates age‐related pathologies including osteoporosis (Guo et al. 2021), atherosclerosis (Wang and Bennett 2012; Xiang et al. 2022), and non‐alcoholic fatty liver disease (NAFLD) (Ogrodnik et al. 2017). Accumulating evidence demonstrates that targeted elimination of SNCs can ameliorate age‐related tissue dysfunction and extend health span in preclinical models (Baker et al. 2011). This has spurred the development of senolytic drugs designed to selectively clear SNCs. First‐generation senolytics, such as the dasatinib plus quercetin (DQ) combination, have shown efficacy in reducing SNC burden and improving physiological function across multiple aging and disease contexts (Chaib et al. 2022). Other senolytic strategies include small‐molecule inhibitors like ABT263 (navitoclax) and ABT737, which induce SNCs apoptosis by targeting the anti‐apoptotic proteins BCL‐2 and BCL‐xL (Khan et al. 2019; Tailler et al. 2019), as well as the prodrug SSK1, which exploits elevated lysosomal β‐galactosidase (β‐gal) activity in SNCs to release cytotoxic payloads (Cai et al. 2020). Innovative approaches like CAR‐T cells targeting SNCs surface markers have also shown promise in restoring tissue homeostasis in murine models (Amor et al. 2020; Yang et al. 2023). Despite this progress, significant challenges remain. Clinical translation of senolytics is hindered by drug‐specific limitations: ABT263 causes transient thrombocytopenia and neutropenia (Murakami et al. 2022); SSK1 exhibits off‐target toxicity due to basal β‐gal activity in immune cells (Cai et al. 2020); and CAR‐T therapies risk cytokine release syndrome (Amor et al. 2020; Balagopal et al. 2022). Moreover, the heterogeneity of senescence phenotypes across tissues leads to variable drug sensitivities among SNCs subpopulations, limiting the broad‐spectrum utility of current therapies (Cai et al. 2020; Zhu et al. 2016). These issues underscore the unmet need for safer, more universal strategies to target diverse SNCs in aging organisms.

While current senolytic strategies primarily target apoptotic pathways or surface markers, the metabolic dependencies distinguishing SNCs from normal cells remain underexplored. A promising metabolic vulnerability involves asparagine (Asn) homeostasis, regulated by asparagine synthetase (ASNS), the sole enzyme synthesizing Asn intracellularly (Sun et al. 2023). Clinically, the asparagine‐depleting enzyme L‐asparaginase (ASNase) exploits this pathway to treat ASNS‐deficient cancers, including acute lymphoblastic leukemia (ALL), by hydrolyzing extracellular Asn into aspartate and ammonia (Richards and Kilberg 2006). ASNS‐deficient tumor cells, unable to synthesize Asn *de novo*, undergo proliferation arrest and apoptosis upon Asn deprivation (Akahane et al. 2022; Chiu et al. 2019), a mechanism leveraged in treating hematologic malignancies such as ALL, acute myeloid leukemia (Takahashi et al. 2012), non‐Hodgkin lymphoma (Kobrinsky et al. 2001), and multiple myeloma (Agrawal et al. 2003). Here, we identify ASNS downregulation as a metabolic hallmark of SNCs across multiple senescence models. Analogous to ASNS‐deficient tumors, SNCs exhibit heightened susceptibility to Asn depletion due to their impaired biosynthetic capacity. We demonstrate that ASNase‐mediated Asn restriction selectively eliminates SNCs, mitigates age‐related functional decline, and delays the progression of aging‐associated pathologies in mice.

## 2 Results

### 2.1 ASNS Deficiency Characterizes Cellular Senescence and Tissue Aging

**FIGURE 1:**
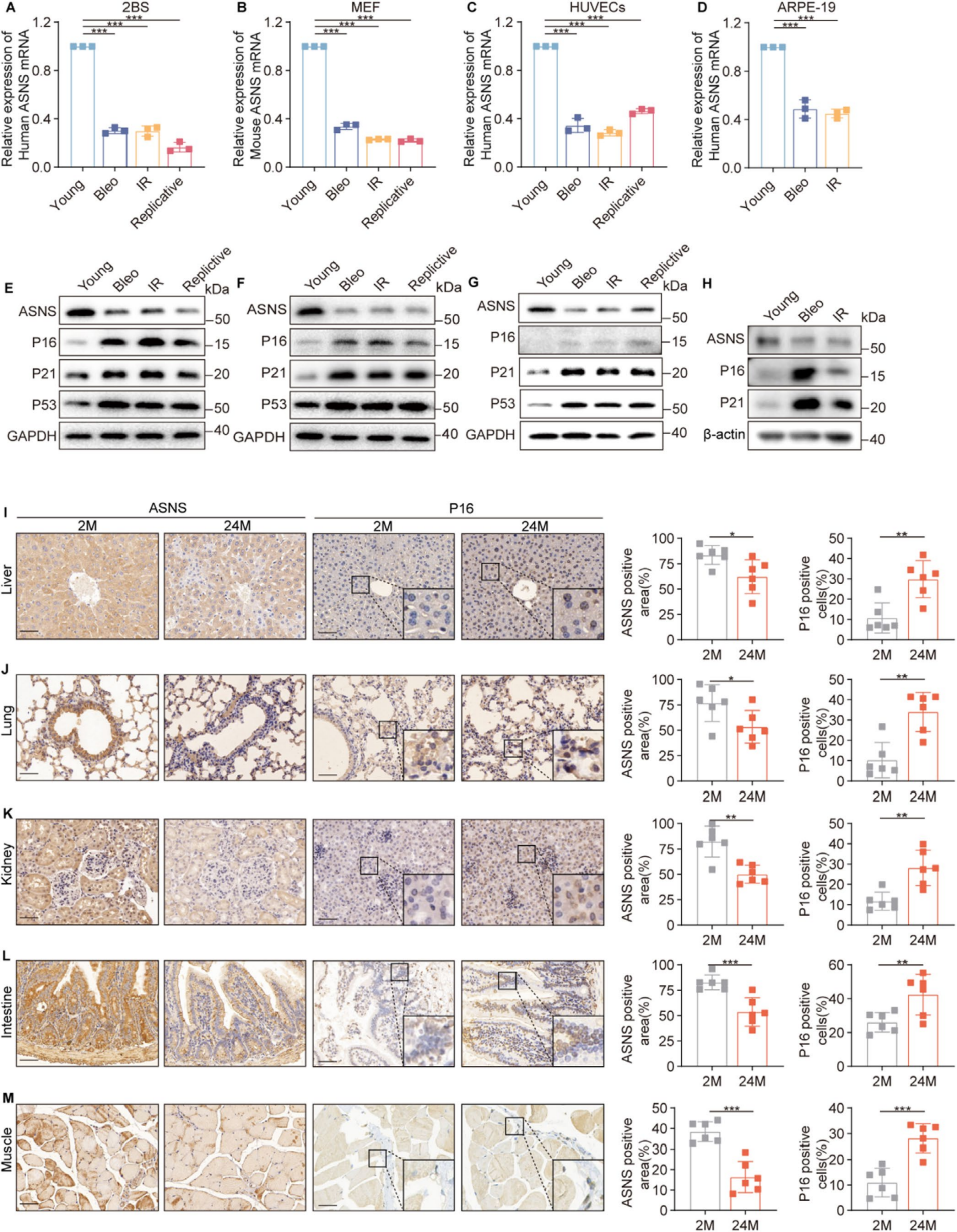
ASNS expression is reduced in senescent cells and tissues of aged mice. (A–D) Detection of ASNS mRNA in bleomycin‐induced prematurely senescent cells(Bleo), ionizing radiation‐induced prematurely senescent cells(IR) and replicatively senescent cells(Replicative) by RT‐qPCR, with young cells as a control and GAPDH as the internal control.(*n* = 3 per group). (A) Human fetal lung diploid fibroblast cell line(2BS), (B) Mouse Embryonic Fibroblast (MEF), (C) Primary human umbilical vein endothelial cells (HUVECs), (D) Adult Retinal Pigment Epithelial cell line‐19(ARPE‐19). (E–H) Detection of ASNS protein and senescence‐associated protiens (P53, P21, P16) in bleomycin‐induced prematurely senescent cells(Bleo), ionizing radiation‐induced prematurely senescent cells(IR) and replicatively senescent cells (Replicative) by western blot. (E) Human fetal lung diploid fibroblast cell line(2BS), (F) Mouse Embryonic Fibroblast (MEF), (G) Primary human umbilical vein endothelial cells (HUVECs). (H) Adult Retinal Pigment Epithelial cell line‐19(ARPE‐19). (I–M) Representative staining and quantification of ASNS‐positive area and P16‐positive cells of 2‐month‐old (2 M) and 24‐month‐old‐mice (24 M) by IHC (*n* = 6 mice per group). Scale bar, 50 μm. (I) Liver, (J) Lung, (K) Kidney, (L) Intestine, (M) Muscle. Data are presented as mean ± SEM. Unpaired two‐tailed *t* test for (I–M), one‐way ANOVA test for (A–D), *p < 0.05, **p < 0.01, ***p < 0.001. ns, not significant.

ASNS, the sole enzyme catalyzing Asn biosynthesis, mediates the ATP‐dependent conversion of aspartate and glutamine into Asn and glutamate (Cai et al. 2022). To investigate whether SNCs exhibit dysregulated asparagine metabolism, we first analyzed ASNS expression across multiple senescence models. In human fetal lung diploid fibroblasts (2BS), ASNS mRNA levels were significantly reduced in SNCs induced by genotoxic stress (bleomycin treatment), ionizing radiation (IR), and replication stress, compared to young proliferating controls (Figure 1A). This downregulation was evolutionarily conserved, with mouse embryonic fibroblasts (MEF) showing similar ASNS suppression during senescence (Figure 1B). The phenomenon extended to diverse cell types, including human umbilical vein endothelial cells (HUVECs) and adult retinal pigment epithelial cells (ARPE‐19), which exhibited marked ASNS mRNA reduction upon senescence (Figure 1C,D). Western blot analysis confirmed corresponding decreases in ASNS protein levels across all tested SNC models (Figure 1E–H). Transcriptomic data from Aging Atlas (https://ngdc.cncb.ac.cn/aging/index) further validated ASNS downregulation in IR‐induced and replicative senescent human diploid fibroblasts (WI38, IMR90) and arterial endothelial cells (HAECs) (Figure S1A–C). Importantly, intracellular Asn levels were correspondingly depleted in these SNCs (Figure S2A–D), functionally linking reduced ASNS activity to asparagine deficiency.

To assess physiological relevance, we examined ASNS expression in aged mouse tissues. Liver, lung, kidney, intestine, and muscle from aged mice showed significantly increased P16 protein levels (Figure 1I–M), confirming elevated senescent cell burden. Strikingly, these tissues exhibited corresponding decreases in ASNS expression (Figure 1I–M), mirroring our in vitro findings. These results establish ASNS deficiency as a consistent feature of both cellular senescence and tissue aging.

Transcription factor ATF4, a known master regulator of ASNS, typically induces ASNS expression through the amino acid response (AAR) and unfolded protein response (UPR) pathways during nutrient stress (Lomelino et al. 2017). Surprisingly, despite the evidence of partial AAR/UPR pathway activation (elevated p‐GCN2 and p‐eIF2α; Figure S3A,B), both ATF4 mRNA and protein levels were significantly reduced in SNCs. ATF4 overexpression in senescent cells restored ASNS mRNA and protein expression (Figure S3D,E); furthermore, computational prediction using JASPAR identified two ATF4 binding sites within the ASNS promoter region, both exhibiting high‐affinity potential with relative profile scores exceeding 0.9 (Figure S3F,G), demonstrating that ATF4 deficiency is responsible for ASNS downregulation during senescence.

### 2.2 ASNS Influences Cellular Senescence Through Asn Metabolism

**FIGURE 2:**
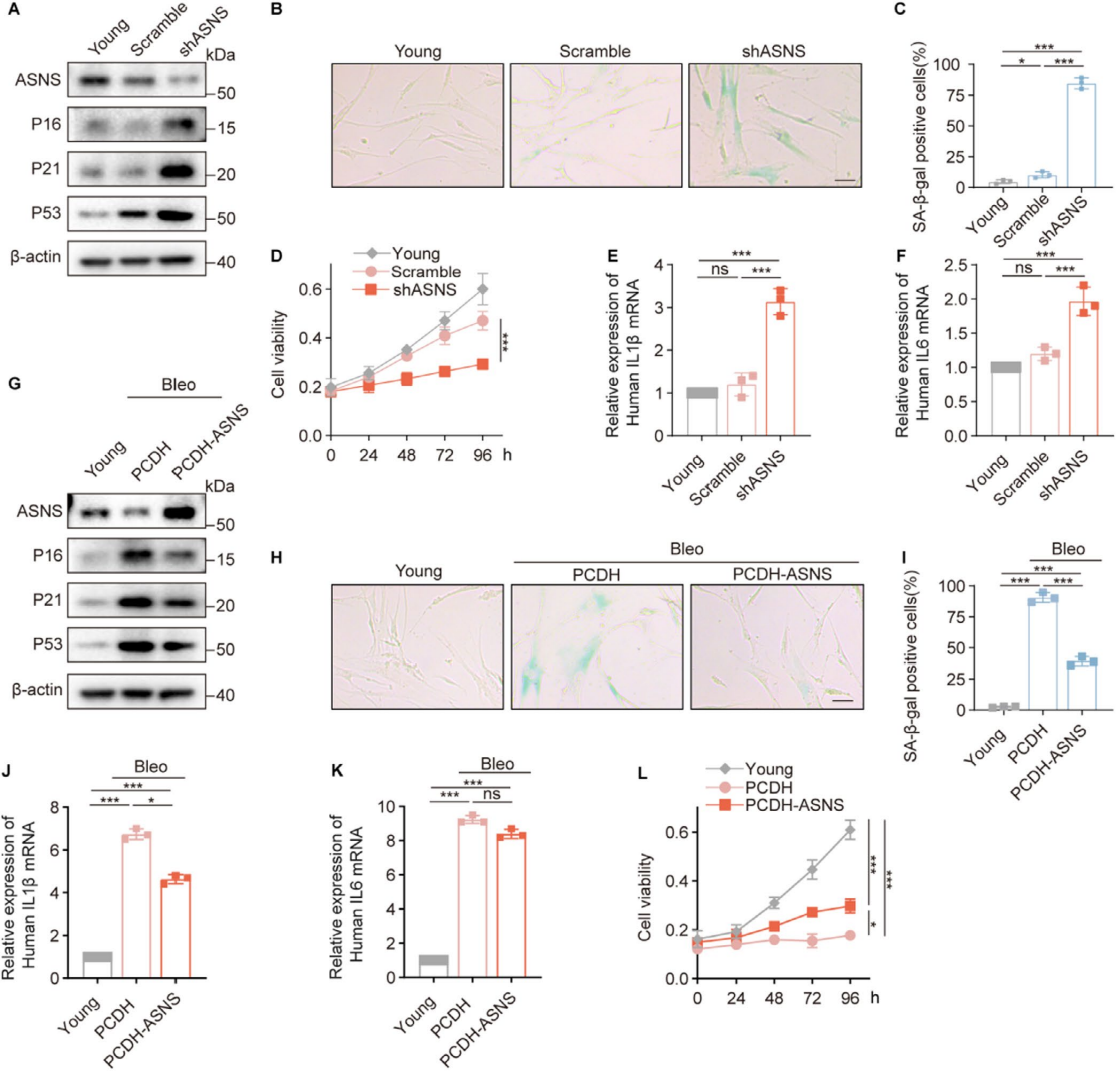
ASNS influences cellular senescence through Asn. (A–F) Detection of senescence‐associated markers in wild‐type young 2BS cells and 2BS cells infected with lentivirus containing either the pLKO.1‐scramble (nonsense shRNA sequence) or pLKO.1‐shASNS. (A) Detection ASNS protein and senescence‐associated protiens. (B) Representative images and (C) quantification of SA‐β‐gal staining (*n* = 3). Scale bar, 50 μm. (D) Quantification of viability of cells (*n* = 3). (E, F) Quantification of IL1β and IL6 genes by RT‐qPCR. (G–L) Detection of senescence‐associated markers in wild‐type young 2BS cells and 2BS cells treated with bleomycin for 7 days, following infection with lentivirus containing either the PCDH vector or the PCDH‐ASNS vector. (G) Detection ASNS protein and senescence‐associated protiens. (H) Representative images and (I) quantification of SA‐β‐gal staining (*n* = 3). Scale bar, 50 μm. (J, K) Quantification of IL1β and IL6 genes by RT‐qPCR. (L) Quantification of viability of cells (*n* = 3). Data are presented as mean ± SEM. One‐way ANOVA test for (C, E, F, I–K), two‐way ANOVA test for (D, L), *p < 0.05, ***p < 0.001. ns, not significant.

To elucidate the functional role of ASNS in cellular senescence, we first examined the consequences of ASNS depletion in young cells. Knockdown of ASNS significantly elevated senescence markers, including P16, P21, and P53 protein levels (Figure 2A), increased SA‐β‐gal activity (Figure 2B,C), and enhanced secretion of SASP factors IL1β and IL6 (Figure 2E,F), while concurrently suppressing cellular proliferation (Figure 2D). These results demonstrate that ASNS deficiency alone is sufficient to trigger premature senescence. Complementary to these loss‐of‐function experiments, we overexpressed ASNS in young 2BS fibroblasts prior to inducing senescence with bleomycin. Assessment at day 7 post‐treatment revealed that ASNS overexpression mitigated bleomycin‐induced increases in P16, P21, and P53 protein levels (Figure 2G), reduced the percentage of SA‐β‐gal positive cells (Figure 2H,I), and decreased IL1β expression (Figure 2J,K). While ASNS‐overexpressing cells maintained proliferative capacity for 7 days after bleomycin exposure, subsequent monitoring revealed proliferation arrest occurring at 72 h post this initial period (Figure 2L). This temporal pattern indicates that elevated ASNS expression can delay but not reverse the established senescence.

Given that Asn and Glu are direct catalytic products of ASNS activity, we next investigated whether the senescence phenotype induced by ASNS knockdown stems from metabolic perturbation. Intracellular metabolite analysis revealed a significant reduction in Asn levels in ASNS‐deficient cells, while Glu concentration remained unchanged (Figure S4A,B). To determine the functional specificity of these metabolites, we performed rescue experiments with exogenous supplementation. Notably, Glu supplementation showed no significant effects on ASNS knockdown‐induced senescence markers, including P16/P21/P53 protein levels (Figure S4C), proliferation rate (Figure S4D), or SA‐β‐gal positivity (Figure S4E,F). In contrast, Asn supplementation effectively normalized all these senescence parameters (Figure S4C–F), demonstrating the specific requirement for Asn in maintaining cellular homeostasis.

To further define the temporal requirements for asparagine in senescence progression, we supplemented Asn at different time points following bleomycin treatment. Early Asn administration (day 0) significantly mitigated the upregulation of P16, P21, and P53 (Figure S4H), decreased SA‐β‐gal‐positive cells (Figure S4I,J), and maintained cell growth (Figure S4K). In contrast, delayed Asn supplementation (day 5) showed no protective effects (Figure S4H–K), suggesting that Asn primarily functions to slow senescence initiation rather than reverse its progression.

Collectively, these results establish ASNS as a critical metabolic regulator of cellular senescence through its control of asparagine biosynthesis. Our data demonstrate that ASNS deficiency drives premature senescence via Asn depletion, while its overexpression delays but cannot abrogate the senescent state. Mechanistically, this regulation depends specifically on ASNS‐derived asparagine production, highlighting a previously unrecognized metabolic checkpoint in senescence control.

### 2.3 ASNase and Autophagy Inhibition Synergistically Eliminate Senescent Cells

Building upon our observation of reduced ASNS expression and intracellular asparagine (Asn) levels in senescent cells (Figure S2A–D), we sought to determine whether metabolic targeting of Asn could selectively eliminate senescent cells (SNCs). Treatment with asparaginase (ASNase) at concentrations ranging from 0.5 to 3 U/mL effectively depleted extracellular Asn (Figure S5A) and induced dose‐dependent apoptosis in SNCs, with increases ranging from 8% to 22% (Figure S5B,C). Notably, young cells exhibited minimal apoptosis except at the highest concentration tested (3 U/mL), suggesting a potential therapeutic window at lower doses. High concentrations of ASNase might cause nonspecific cell apoptosis. Thus, 0.5 U/mL ASNase was selected for the following experiments. However, residual intracellular Asn persisted in ASNase‐treated SNCs (Figure S5D), prompting investigation into alternative Asn sources that might sustain cell survival under conditions of extracellular deprivation.

**FIGURE 3:**
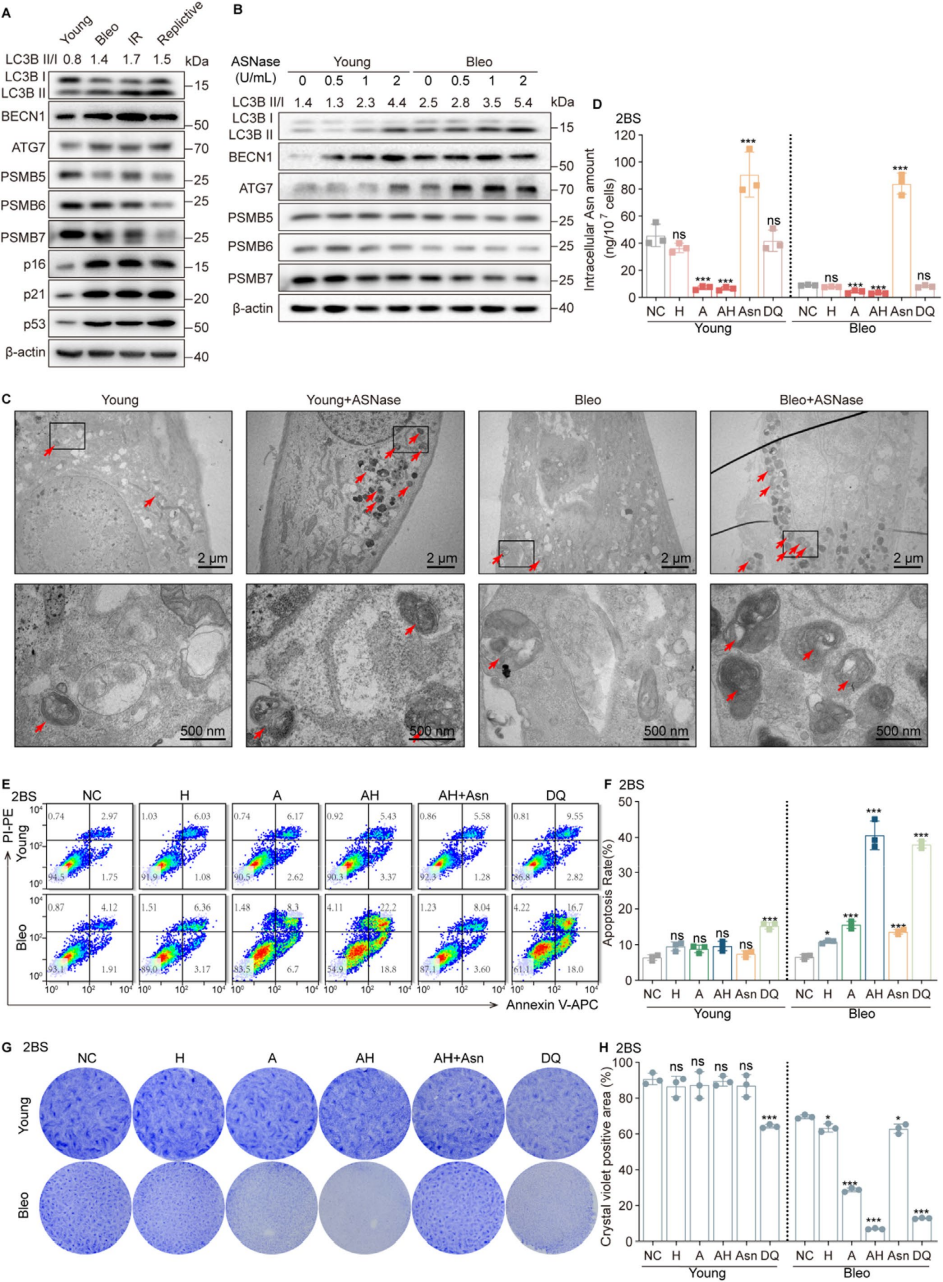
ASNase and autophagy inhibition promote apoptosis in senescent cells by limiting asparagine availability. (A) Detection of autophagy‐related proteins, proteasome‐related proteins, and senescence‐associated proteins by western blot in young and senescent cells. (B) Detection of autophagy‐related proteins and proteasome‐related proteins by western blot in young and senescent cells following ASNase treatment (0.5 U/mL, 3 days). (C) Transmission electron microscopy (TEM) analysis of autophagic ultrastructures in ASNase‐treated young and senescent cells (0.5 U/mL, 3 days). (D–H) Young and senescent cells were subjected to the following treatments: “NC” indicates the negative control, “H” indicates cells treated with HCQ (25 μM, 3 days), “A” indicates treatment with ASNase (0.5 U/mL, 3 days), “AH” indicates simultaneous treatment with both ASNase and HCQ, “AH + Asn” or “Asn” indicates treatment with Asn (200 μM) with replenishment every 12 h after “AH” treatment, and “DQ” indicates treatment with dasatinib (0.25 μM) and quercetin (50 μM). (D) Detection of intracellular Asn levels by LC–MS/MS, (E) detection of cell apoptosis by flow cytometry and (F) analysis of apoptosis rate. (G) Crystal violet staining and (H) analysis of crystal violet staining positive area. Data are presented as mean ± SEM. One‐way ANOVA test for (D, F, H), *p < 0.05, ***p < 0.001. ns, not significant.

In mammalian cells, cellular Asn pools are maintained through three primary pathways: extracellular uptake, *de novo* synthesis via ASNS, and protein degradation‐mediated recycling (Davidsen and Sullivan 2020; Hinze et al. 2019). Given the observed downregulation of ASNS in senescent cells and our pharmacological blockade of extracellular Asn with ASNase, we hypothesize that protein degradation might compensate to maintain Asn levels. Protein degradation in mammalian cells occurs primarily through two major pathways: the ubiquitin–proteasome system and the autophagy–lysosomal pathway. Our comparative analysis revealed that senescent cells exhibited decreased expression of proteasome subunits (PSMB5, PSMB6, PSMB7) but upregulated key autophagy regulators (BECN1, ATG7) and increased LC3BII/I ratios (Figure 3A). More importantly, following ASNase treatment, the expression of autophagy‐related proteins was elevated (Figure 3B). Electron microscopy revealed no obvious difference in autophagosome abundance between untreated young and senescent cells. However, ASNase treatment induced marked autophagosome accumulation in both young and senescent cells (Figure 3C). This suggests that under conditions of asparaginase‐induced decline in Asn levels, senescent cells may increase autophagic flux to maintain cellular Asn requirements, which is consistent with other reports (Gamerdinger et al. 2009; Wang et al. 2012; Young et al. 2009; Zhang et al. 2023).

To functionally assess the role of autophagy in senescent cell survival following ASNase treatment, we generated stable cell lines with knockdown of essential autophagy genes (BECN1/ATG7) (Figure S6A,B). Genetic inhibition of autophagy synergized with ASNase treatment to further deplete intracellular Asn levels (Figure S6C) and significantly enhance apoptosis compared to ASNase alone (Figure S6D,E). The specificity of this effect was confirmed by complete rescue with exogenous Asn supplementation (Figure S6C–E). We extended these findings using the clinically approved autophagy inhibitor hydroxychloroquine (HCQ) (Figure S7A–D), demonstrating that the ASNase + HCQ (AH) combination potently reduced intracellular Asn (Figure 3D) and increased senescent cell apoptosis by approximately 40% (Figure 3E–H). Strikingly, this effect was highly selective for senescent cells, whereas the established senolytic cocktail dasatinib + quercetin (DQ) induced comparable apoptosis in both young and senescent populations (Figure 3E–H). Mechanistically, analogous to tumors under nutrient stress (Darnal et al. 2024; Takahashi et al. 2017), AH triggers mitochondrial apoptotic cascades in senescent cells: immunoblotting detected decreased anti‐apoptotic BCL‐XL, increased pro‐apoptotic Bax, and elevated activated caspase‐9 and caspase‐3 (Figure S8A). This demonstrates that AH induces senescent cell death via the BCL‐2 family‐mediated mitochondrial apoptosis pathway.

The therapeutic potential of this approach was further validated across multiple senescence models, including replicative senescent HUVECs (Figure S10A–E), IR‐induced senescent 2BS cells (Figure S11A–C), replicative senescent 2BS cells (Figure S11D–F), bleomycin‐induced ARPE‐19 cells (Figure S11G–I), and replicative senescent MEFs (Figure S11J–L). In all models, AH treatment demonstrated consistent efficacy in eliminating SNCs while sparing proliferating counterparts.

Prompted by these findings, we investigated AH's potential in eliminating therapy‐induced senescent (TIS) tumor cells—a state arising during chemo/radiotherapy that enables tumor survival under sublethal stress (Basu 2022). TIS is characterized by senescence phenotypes, including elevated levels of senescence‐associated proteins, increased secretion of SASP, and proliferation arrest. TIS is believed to help tumor cells evade death during sublethal drug therapy. Under certain conditions, TIS can reactivate proliferative potential and lead to cancer recurrence (Saleh et al. 2020). Therefore, eliminating TIS cells has become an important area in the field of cancer research.

**FIGURE 4:**
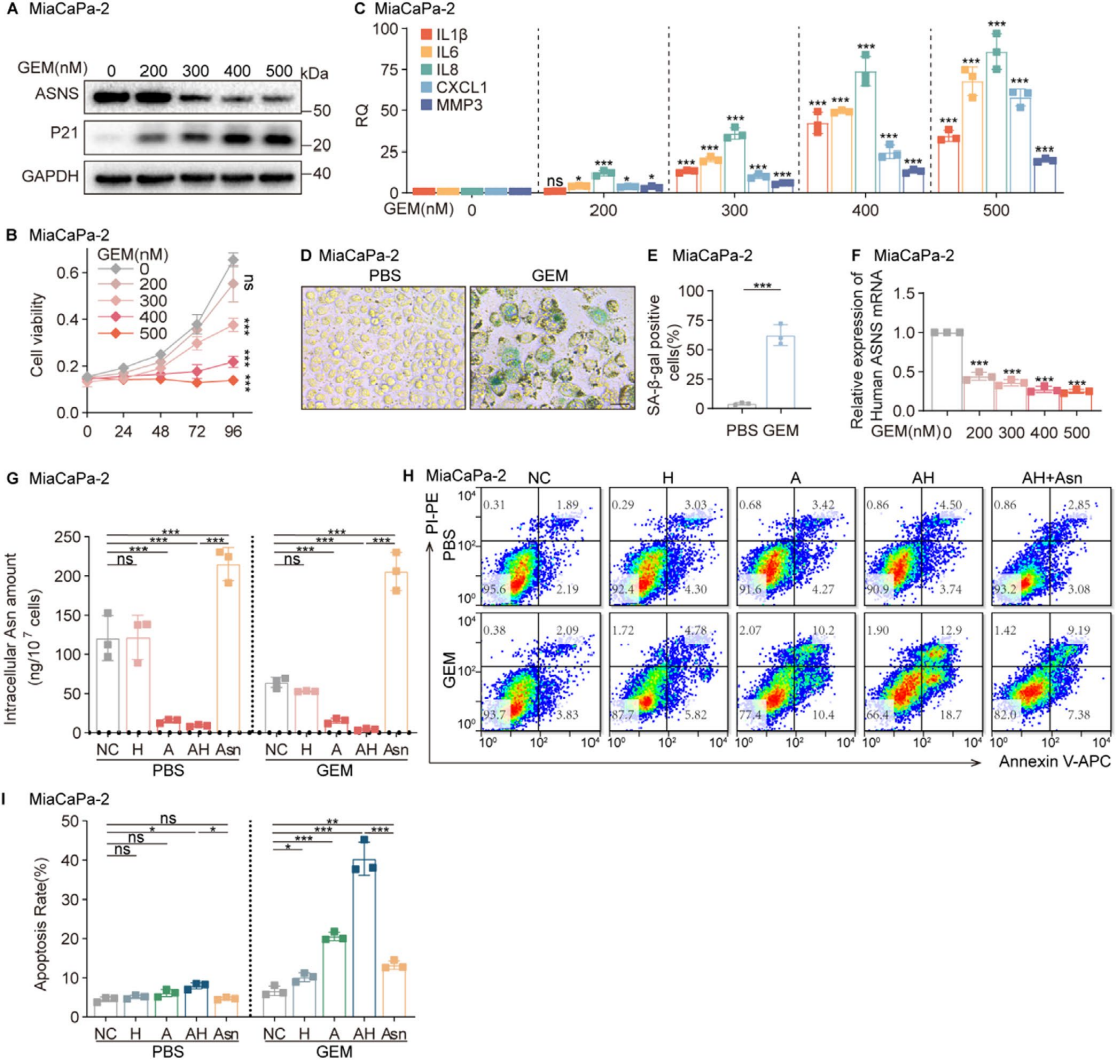
AH eliminates TIS tumor cells by limiting asparagine availability. (A) Detection of ASNS and senescence‐associated protein P21 in pancreatic cancer cells MiaCaPa‐2 treated with gemcitabine (0–500 nM, 5 days). (B) Quantification of viability of MiaCaPa‐2 cells (*n* = 3). (C) Detection of SASP mRNA in MiaCaPa‐2 cells. (D) Representative images and (E) quantification of SA‐β‐gal staining of MiaCaPa‐2 cells treated with gemcitabine (500 nM, 5 days) (*n* = 3). Scale bar, 50 μm. (F) Detection of ASNS mRNA in MiaCaPa‐2 cells treated with gemcitabine (0–500 nM, 5 days). (G–I) MiaCaPa‐2 cells treated with gemcitabine (500 nM, 5 days) were subjected to the following treatments: “NC” indicates the negative control, “H” indicates cells treated with HCQ (25 μM, 3 days), “A” indicates treatment with ASNase (0.5 U/mL, 3 days), “AH” indicates simultaneous treatment with both ASNase and HCQ, “AH + Asn” or “Asn” indicates treatment with Asn (200 μM) with replenishment every 12 h after “AH” treatment (*n* = 3 per group). (G) Detection of intracellular Asn levels by LC–MS/MS. (H) Detection of cell apoptosis by flow cytometry and (I) analysis of apoptosis rate. Data are presented as mean ± SEM. Two‐way ANOVA test for (B), unpaired two‐tailed *t* test for (E), one‐way ANOVA test for (C, F, G, I), *p < 0.05, **p < 0.01, ***p < 0.001. ns, not significant.

We subsequently explored the translational relevance of these findings in TIS using gemcitabine‐treated pancreatic cancer cells (MiaPaCa‐2). Cells undergoing TIS exhibited characteristic features including P21 upregulation (Figure 4A), proliferation arrest (Figure 4B), increased SASP secretion (Figure 4C), and elevated SA‐β‐gal activity (Figure 4D,E). Mirroring our observations in normal SNCs, these senescent cancer cells showed significant ASNS downregulation at both mRNA (Figure 4F) and protein levels (Figure 4A), rendering them susceptible to AH‐mediated Asn depletion (Figure 4G) and subsequent apoptosis (Figure 4H,I). Parallel results in PANC‐1 cells (Figure S12A–I) confirmed the broad applicability of this metabolic vulnerability across cellular contexts.

### 2.4 AH Treatment Eliminates SNCs and Improves Physical Function in Aged Mice

**FIGURE 5:**
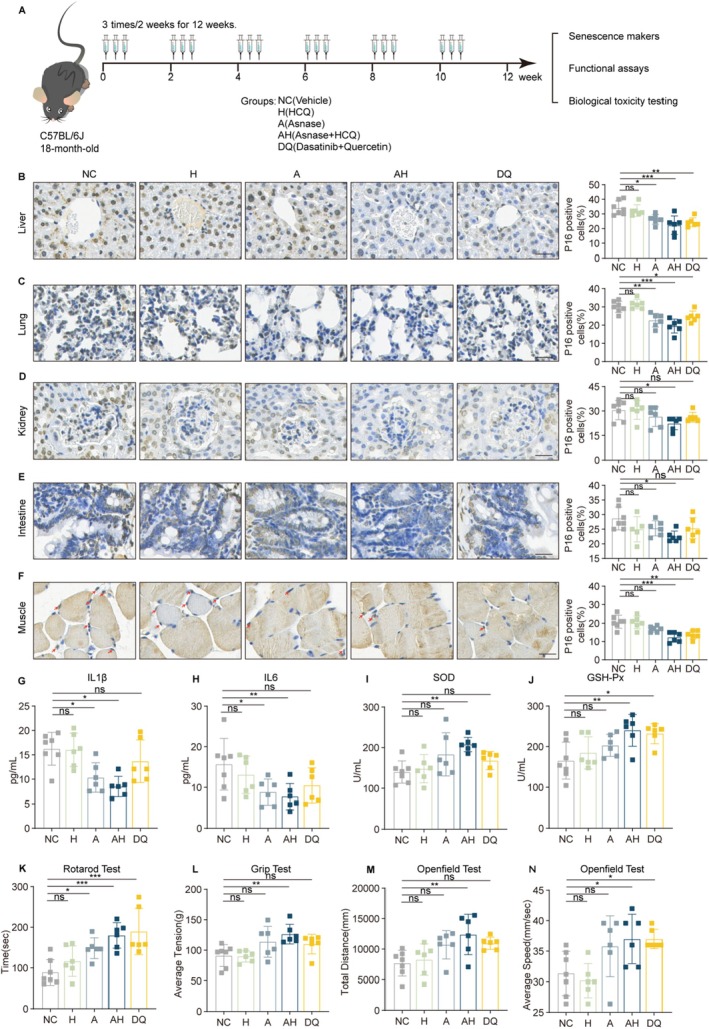
AH deletes senescent cells and improves physical function of aged mice. A. Experimental design for AH treatment of aged mice. Eighteen‐month‐old mice were divided into the following groups and treated every 2 weeks for three consecutive days over a period of 12 weeks. NC group (*n* = 7): Intraperitoneal injection of vehicle (saline) and oral gavage of vehicle (4% DMSO +10% PEG400 + 86% ddH2O). HCQ group (*n* = 6): Intraperitoneal injection of vehicle and oral gavage of HCQ (40 μg/g). ASNase group (*n* = 6): Intraperitoneal injection of ASNase (2 U/g) and oral gavage of vehicle. AH group (*n* = 6): Intraperitoneal injection of ASNase (2 U/g) and oral gavage of HCQ (40 μg/g). DQ group (*n* = 6): Oral gavage of dasatinib (5 μg/g) and quercetin (50 μg/g). B–F. Representative images (left) and quantification (right) of P16‐positive cells by IHC. (B) Liver, (C) Lung, (D) Kidney, (E) Intestine, (F) Muscle. Scale bars, 50 μm. (G, H) Detection of SASP proteins in serum by ELISA. (G) IL1β and (H) IL6. I‐J. Detection of SOD (I) and GSH‐Px (J) in serum. (K) Quantification of maximal time on rotarod. (L) Grip strength of four limbs of aged mice. (M) Total distance of aged mice in openfield test. (N) Average speed of aged mice in openfield test. Data are presented as mean ± SEM. One‐way ANOVA test for (B–N), *p < 0.05, **p < 0.01, ***p < 0.001. ns, not significant.

To evaluate the therapeutic potential of AH in vivo, we treated 18‐month‐old C57BL/6J mice (a naturally aged mouse model) with ASNase (intraperitoneal injection) and HCQ (oral gavage) for 12 weeks (Figure 5A). Biochemical analysis confirmed that ASNase treatment effectively reduced serum Asn levels (Figure S14A). Immunohistochemical evaluation revealed that while HCQ monotherapy showed minimal effects on P16‐positive cell clearance, the ASNase or AH combination significantly reduced senescent cell burden across multiple tissues, including liver, lung, kidney, small intestine, and muscle (Figure 5B–F). This senolytic efficacy was further supported by complementary senescence markers: decreased SA‐β‐gal staining (Figure S13A–E) and increased LaminB1 expression (Figure S13F–J) in tissues from AH‐treated animals. These findings indicate that AH intervention effectively clears senescent cells in tissues. In addition, AH treatment reduced the serum levels of IL1β and IL6 (Figure 5G,H). Oxidative/antioxidative system imbalance has been widely used as a hallmark of aging (Cand and Verdetti 1989). After AH treatment, serum antioxidant markers superoxide dismutase (SOD) (Figure 5I) and glutathione peroxidase (GSH‐Px) (Figure 5J) significantly increased, with no notable changes observed in the lipid oxidation marker malondialdehyde (MDA) (Figure S14B). These results indicated that AH enhanced the antioxidative potential of aged mice.

Functional assessment demonstrated comprehensive physiological improvements in AH‐treated animals. These mice exhibited significantly enhanced motor performance in rotarod testing (Figure 5K), increased grip strength (Figure 5L, Figure S14C), and greater exploratory behavior in open field assays (Figure 5M,N, Figure S14D–G). The concordance between senescent cell clearance, reduced inflammation, improved oxidative stress markers, and enhanced physical function provides compelling evidence that AH combination therapy represents a promising strategy for mitigating multiple aspects of age‐related functional decline.

### 2.5 AH Treatment Attenuates Progression of Senescence‐Associated Diseases

Given the established role of SNCs in age‐related pathologies, we evaluated the therapeutic potential of AH combination therapy in three prototypical senescence‐associated disease models: senile osteoporosis (SOP), non‐alcoholic fatty liver disease (NAFLD), and atherosclerosis.

**FIGURE 6:**
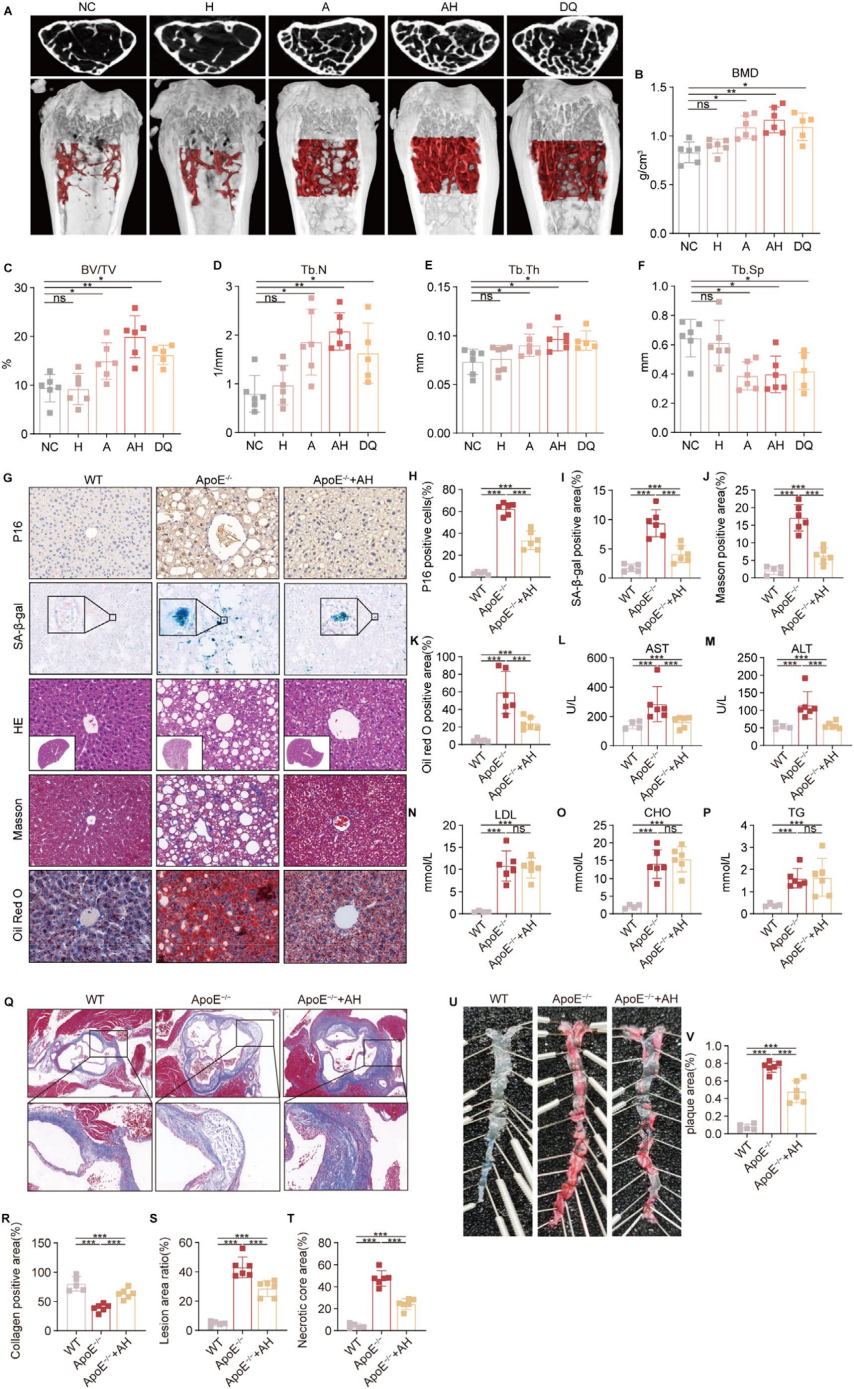
AH delays the progression of senescence‐associated diseases. (A–F) Four‐month‐old SAMP/6 mice were divided into the following groups and treated every 2 weeks for three consecutive days over a period of 12 weeks. NC group (*n* = 6): Intraperitoneal injection of vehicle (saline) and oral gavage of vehicle (4% DMSO +10% PEG400 + 86% ddH2O). HCQ group (*n* = 6): Intraperitoneal injection of vehicle and oral gavage of HCQ (40 μg/g). ASNase group (*n* = 6): Intraperitoneal injection of ASNase (2 U/g) and oral gavage of vehicle. AH group (*n* = 6): Intraperitoneal injection of ASNase (2 U/g) and oral gavage of HCQ (40 μg/g). DQ group (*n* = 6): Oral gavage of dasatinib (5 μg/g) and quercetin (50 μg/g). (A) Micro‐CT analysis of bone density and trabecular structure in the distal femur of mice. The top images show cross‐sections of the distal femur, and the bottom images show 3D reconstructions with the red region indicating the ROI. (B) Bone mineral density (BMD), (C) bone volume to tissue volume ratio (BV/TV), (D) trabecular number (Tb.N), (E) trabecular thickness (Tb.Th), and (F) trabecular separation (Tb.Sp). (G–V) Two‐month‐old C57BL/6J‐ApoE^−/−^ and C57BL/6J wild‐type mice were divided into the following groups and treated every 2 weeks for three consecutive days over a period of 12 weeks. WT group: C57BL/6J wild‐type mice (*n* = 6). ApoE^−/−^ group (*n* = 6): C57BL/6J‐ApoE^−/−^ mice fed a high‐fat diet, intraperitoneal injection of vehicle (saline), and oral gavage of vehicle (4% DMSO +10% PEG400 + 86% ddH2O). ApoE^−/−^ + AH group (*n* = 6): C57BL/6J‐ApoE^−/−^ mice fed a high‐fat diet, intraperitoneal injection of ASNase (2 U/g), and oral gavage of HCQ (40 μg/g). (G) Histological analysis of liver tissues in mice, IHC detection of P16 protein, and (H) quantification of P16‐positive cells, SA‐β‐gal staining and (I) quantification of SA‐β‐gal positive rate, HE staining, Masson staining and (J) quantification, Oil Red O staining and (K) quantification, scale bar, 50 μm. (L–P) Serum biochemical tests. The levels of (L) aspartate transaminase (AST), (M) alanine transaminase (ALT), (N) low‐density lipoprotein (LDL), (O) cholesterol (CHO), (P) triglycerides (TG). (Q) Masson staining of the aortic root, (R) quantification of collagen area in the aortic root, (S) quantification of lesion area in the aortic root, scale bar, 50 μm, (T) quantification of necrotic core area in the aortic root. (U) Oil Red O staining images from the aortic arch to the iliac artery and (V) quantification of atherosclerotic plaque area. Data are presented as mean ± SEM. One‐way ANOVA test for (B–F, H–P, R–T, V), *p < 0.05, **p < 0.01, ***p < 0.001. ns, not significant.

SOP, characterized by progressive bone mass reduction and microstructural deterioration (Ali et al. 2024), represents a prototypical age‐related disorder where senescent cell accumulation contributes to pathogenesis (Farr et al. 2017; Guo et al. 2021). Using 4‐month‐old male SAM‐P/6 mice (Figure S15A)—a well‐characterized model of age‐related bone loss (Matsushita et al. 1986)—we evaluated the therapeutic potential of AH treatment. Three months of AH treatment significantly reduced P16‐positive cells in the bone marrow of the right femur compared to the control group (Figure S15B,C). Micro‐CT analysis of the left femur (Figure 6A) revealed differential treatment effects: while HCQ monotherapy showed minimal impact on bone mineral density (BMD), both ASNase (A) and AH combination therapy significantly increased BMD, with AH demonstrating superior efficacy (Figure 6B). The established senolytic cocktail DQ (dasatinib + quercetin) also enhanced BMD, though to a lesser degree than AH treatment. Comprehensive trabecular bone analysis showed that AH treatment improved multiple microstructural parameters, including increased bone volume/tissue volume ratio (BV/TV; Figure 6C), greater trabecular number (Tb.N; Figure 6D), enhanced trabecular thickness (Tb.Th; Figure 6E), and reduced trabecular spacing (Tb.Sp; Figure 6F). Biochemical assessment revealed decreased serum alkaline phosphatase levels in AH‐treated mice (Figure S15D), consistent with reduced bone turnover, while calcium and phosphorus homeostasis remained unaffected (Figure S15E,F). These findings demonstrate that AH combination therapy effectively reduces senescence cell burden in bone tissue and improves multiple parameters of bone quality, supporting its potential as a therapeutic strategy for senile osteoporosis.

To investigate AH's therapeutic effect on NAFLD progression, we employed 12‐week‐old ApoE(−/−) mice maintained on a high‐fat diet for 12 weeks (Figure S16A), a well‐established model for studying NAFLD pathogenesis. Research indicates that SNCs play a crucial role in the onset and progression of NAFLD (Engelmann & Tacke, 2022; Meijnikman et al. 2021). Following 12 weeks of AH treatment, quantitative analysis revealed significant reductions in hepatic P16‐positive cells (Figure 6G first row, H) and SA‐β‐gal activity (Figure 6G second row, I), demonstrating effective clearance of senescent cells in fatty liver tissue. Histopathological evaluation showed marked improvements in liver architecture with AH treatment. Compared to wild‐type controls, ApoE (−/−) mice exhibited characteristic NAFLD features including hepatocyte ballooning, disorganized parenchymal architecture, and cytoplasmic vacuolization (Figure 6G third row, HE staining). AH treatment substantially ameliorated these changes, with only minimal residual hepatocyte swelling observed (Figure 6G third row). Masson's trichrome staining demonstrated that AH treatment reduced the extensive collagen deposition, characteristic of diet‐induced hepatic fibrosis, in the ApoE (−/−) group (Figure 6G fourth row, J). Correspondingly, Oil Red O staining revealed significantly decreased lipid accumulation in AH‐treated animals (Figure 6G fifth row, K). Biochemical analysis showed that AH treatment normalized serum ALT and AST levels (Figure 6L,M), indicating improved hepatic function. Interestingly, while AH treatment improved liver histology and function, it did not significantly alter serum triglyceride (TG), total cholesterol (CHO), or LDL cholesterol levels (Figure 6N–P), suggesting that the therapeutic benefits occur independently of systemic lipid metabolism modulation. These findings collectively demonstrate that AH treatment effectively reduces senescent cell burden in NAFLD, leading to improved liver histopathology and function without affecting circulating lipid profiles.

Atherosclerosis represents a quintessential age‐related vascular pathology characterized by progressive plaque formation, wherein cellular senescence of endothelial cells, vascular smooth muscle cells, and macrophages plays a pivotal role (Xiang et al. 2022). These senescent cells exacerbate disease progression through sustained secretion of SASP factors, including pro‐inflammatory cytokines and matrix metalloproteinases (MMPs) (Sun et al. 2022). In our atherosclerosis model, immunofluorescence analysis of aortic root sections demonstrated that AH treatment significantly reduced P16‐positive senescent cells within plaques (Figure S16B second column, D). Subsequently, the impact of AH treatment on the progression of atherosclerosis was evaluated. Key characteristics of atherosclerosis include lesion area and plaque stability, with vulnerable plaques being prone to rupture and resulting in acute cardiovascular events (Pérez‐Medina et al. 2020). Compared to the control group, AH‐treated mice exhibited an increase in smooth muscle cells in the aortic root, which was presented by α‐SMA expression (Figure S16B sixth column, F), with no significant change in infiltrating macrophages, which was presented by F4/F8 expression (Figure S16B third column, E). Additionally, AH treatment resulted in an increase in collagen content in the aortic root (Figure 6Q,R), while significantly reducing the lesion area (Figure 6S) and necrotic core size (Figure 6 T). Moreover, oil red O staining of the aorta, extending from the aortic arch to the common iliac artery, revealed a marked reduction in the lesion area in AH‐treated mice compared to those in controls (Figure 6 U,V). These results collectively indicate that AH combination therapy (1) effectively eliminates senescent cells from atherosclerotic plaques, (2) limits plaque progression, and (3) enhances features of plaque stability—particularly through increased α‐SMA+ smooth muscle cell content and consequent collagen deposition—thereby potentially reducing risks of plaque rupture and acute cardiovascular events.

The translational potential of AH therapy necessitated comprehensive safety evaluation. In vitro assessment revealed minimal cytotoxicity in mouse mesenchymal stem cells (m‐MSCs), with only a marginal increase in apoptotic cells observed at therapeutic concentrations (Figure S17A). In vivo toxicity studies demonstrated excellent tolerability following 12‐week administration in mice. Serum biochemical analysis showed no significant alterations in hepatic (ALT, AST) or renal (BUN, creatine) function markers across treatment groups (Figure S18A–D). Complete blood count analysis confirmed all hematological parameters, including white blood cells, red blood cells, and platelet indices, remained within normal physiological ranges (Figure S18E–J). Histopathological examination of liver and kidney tissues revealed preserved organ architecture with no evidence of drug‐induced morphological abnormalities (Figure S19A).

The AH combination therapy exhibited a favorable safety profile across all evaluated parameters. Comprehensive toxicological assessment demonstrated: minimal in vitro cytotoxicity in stem cells; preservation of hepatic and renal function markers; maintenance of physiological hematological parameters; and absence of histopathological abnormalities in major organ systems. These collective findings substantiate that prolonged AH administration is not associated with significant systemic toxicity, thereby addressing a critical prerequisite for clinical development of this senolytic approach.

## 3 Discussion

SNCs accumulation in mammalian tissues and organs correlates with age‐related dysfunction. Studies highlighting the benefits of eliminating harmful SNCs have led to many new initiatives in drug discovery and design (Power et al. 2023). Senolytics are a class of drugs that selectively eliminate SNCs and are promising candidates for the prevention and treatment of chronic conditions. Since the initial discovery of senolytics, therapeutic benefits of multiple senolytic compounds have been reported in pre‐clinical models (Hickson et al. 2019; Shetty et al. 2018). Further promising results have been published from the first human trials with DQ, in which the treatment reduced senescence markers in blood, skin, and adipose tissue and improved physical functioning in a small cohort of patients with idiopathic pulmonary fibrosis (Hickson et al. 2019; Justice et al. 2019). Despite these encouraging results, limitations related to activity spectrum, negative side effects, bioavailability, and potency highlight the need for ongoing research into novel senolytics (Chaib et al. 2022; Kirkland and Tchkonia 2020; Lagoumtzi and Chondrogianni 2021). In this study, our results demonstrated that AH can effectively kill SNCs, improve the physical functions of aging mice, and relieve age‐related diseases; thus, it can be regarded as a potential senolytic drug. AH as senolytic may have the following advantages: (1) AH effectively clears a broad spectrum of SNCs independent of cell origin and stimuli as shown in the study results, suggesting that AH can clear SNCs from various tissue sources, thus effectively improving the overall body function in elderly mice. Thus, AH can be applied to more age‐related diseases. (2) AH is highly specific in eliminating SNCs while minimally affecting nonsenescent cells. As noted previously, there is abnormal expression of ASNS in SNCs. ASNase and HCQ block Asn source pathways outside and inside the cell, respectively, resulting in a lack of available Asn in SNCs and ultimately leading to cell apoptosis. Opposingly, young cells have normal ASNS expression; they can perform independent of extracellular and intracellular Asn, which renders them less sensitive to AH treatment. Moreover, targets selected by other senolytics for clearing SNCs are expressed in young cells, indicating that these drugs affect not only SNCs but also young cells, although the impact on young cells is less pronounced. For example, Navitoclax and Nutlin3a have substantial off‐target apoptotic effects on nonsenescent cell types, such as platelets and immune cells (Mahfoudhi et al. 2016; Wilson et al. 2010). (3) ASNase and HCQ are widely utilized in clinical settings (Lopes et al. 2017; Nirk et al. 2020). Compared to the “start from scratch” approach to drug discovery, a considerable advantage of utilizing established compounds is that their pharmacokinetics, pharmacodynamics, side effects, and toxicity in large populations are already well‐documented. Repurposing these drugs for new indications can expedite specific clinical trials, reduce cost and time, and if successfully approved by regulatory agencies, facilitate their more rapid integration into clinical practice (Armando et al. 2020).

Our study establishes ASNS deficiency as a fundamental metabolic vulnerability of SNCs, enabling selective clearance through combined ASNase and HCQ treatment. While our study demonstrates the efficacy of Asn deprivation in selective senescent cell clearance, we acknowledge parallel research on Reversine‐mediated senescence intervention in myoblasts (Rajabian et al. 2023), yet highlight fundamental mechanistic and therapeutic distinctions: Reversine rejuvenates senescent cells through autophagy‐dependent mitochondrial metabolic restoration, whereas our strategy achieves irreversible senescent cell elimination via Asn deprivation. Crucially, ASNase restricts extracellular Asn uptake but triggers compensatory autophagy to generate intracellular Asn—a salvage pathway that sustains senescence survival, necessitating combined autophagy inhibition to deplete Asn pools and induce apoptosis. This defines our pioneering “nutrient deprivation therapy” paradigm, targeting senescent cell‐specific metabolic vulnerabilities distinct from Reversine's metabolic reprogramming approach.

The AH combination demonstrates significant promise for addressing therapy‐induced senescence (TIS), a major contributor to chemotherapy resistance and treatment‐related morbidity (Chibaya et al. 2022; Saleh et al. 2023; Wang et al. 2020). Clinical observations reveal TIS in 31%–66% of tumors following genotoxic therapies (Saleh et al. 2023), with senescent cancer cells exhibiting stem‐like properties that facilitate tumor repopulation (Was et al. 2018). Moreover, TIS is not only confined to malignant and nonmalignant components of tumor tissues but has also been identified in normal (nontransformed) tissues exposed to chemotherapy or radiotherapy (Saleh et al. 2020; Saleh et al. 2023). It has been reported that HCQ may interfere with the ability of senescent lung cancer cells to repopulate following exposure to DNA‐damaging agents (Olszewska et al. 2021); although the mechanism is still unknown, it is possible that this effect was achieved by HCQ‐clearing senescent cancer cells induced by chemotherapeutic drugs. Furthermore, the extensive senescence of normal cells can cause a decline in normal tissue function, which results in severe treatment‐related side effects. Our findings that AH effectively clears both senescent tumor cells and chemotherapy‐treated normal cells (Figure 4) suggest a dual therapeutic advantage: (1) preventing TIS‐mediated cancer recurrence while (2) mitigating chemotherapy‐induced tissue damage (Lafontaine et al. 2021). To further evaluate AH effectiveness, another in vivo project on TIS elimination by AH is currently undergoing in our laboratory.

In fact, some anti‐aging strategies may be related to depriving SNCs of Asn. For example, reducing Asn can also be achieved by dieting. It is well known that dieting has anti‐aging effects, although the mechanism is unclear (Lee et al. 2021; Longo and Anderson 2022). It is likely that dieting restricts Asn intake, which results in a decrease in Asn levels in the blood (Knott et al. 2018). If this is the case, studying whether HCQ enhances the anti‐aging effect of dieting is crucial. In addition, HCQ has been widely used to treat aging‐related diseases, such as osteoporosis (Both et al. 2018), atherosclerosis (Floris et al. 2018; Shi et al. 2019), rheumatoid arthritis (Nirk et al. 2020), COVID‐19 (Gul et al. 2020), among others. It has been shown that HCQ may inhibit toll‐like receptor 9 (TLR9)/nuclear factor κB (NF‐κB), P53, and CXCR4/CXCL12 pathways, which suppress pro‐inflammatory cytokine production to treat autoimmune diseases (Dima et al. 2022). As an autophagy inhibitor, the possibility cannot be ruled out that HCQ inhibits autophagy in SNCs, thereby reducing available Asn and facilitating SNC clearance. On the basis of our experimental findings, it was observed that Asn elimination in the blood by ASNase or dieting may enhance HCQ therapeutic efficacy for these diseases.

The metabolic vulnerability of SNCs stems from their impaired adaptive response to asparagine deprivation. While normal cells activate the GCN2‐eIF2α‐ATF4 axis to upregulate ASNS during amino acid stress (Lomelino et al. 2017), SNCs exhibit paradoxical ATF4 suppression. Despite evidence of partial AAR/UPR pathway activation (elevated p‐GCN2 and p‐eIF2α; Figure S3A,B), both ATF4 mRNA and protein levels were significantly reduced in SNCs. ATF4 overexpression in senescent cells restored ASNS mRNA and protein expression (Figure S3D,E), demonstrating that ATF4 deficiency is responsible for ASNS downregulation during senescence. This defect prevents compensatory ASNS upregulation, rendering SNCs uniquely sensitive to dual asparagine blockade—extracellular depletion by ASNase and intracellular recycling inhibition by HCQ. Our finding that exogenous asparagine rescues AH‐induced apoptosis (Figure 4G–I) confirms the critical role of asparagine restriction in senolytic activity.

Autophagy is a process that maintains intracellular homeostasis by degrading cellular components and is activated under conditions of nutrient deficiency or other cellular stresses. The role of autophagy in senescence is controversial: autophagy exerts protective functions in organismal aging and age‐related diseases, with its overall activity declining with age; activating autophagy ameliorates age‐related pathologies—for example, spermidine mimics calorie restriction to extend lifespan and alleviate age‐related diseases like cardiovascular disorders, neurodegeneration, and cancer through autophagy‐dependent mechanisms (Madeo et al. 2018), and rapamycin inhibits the mTOR pathway to activate autophagy, extending lifespan in model organisms (e.g., Drosophila, mice) and delaying age‐related diseases (Schinaman et al. 2019). However, the relationship between autophagy and senescent cells is more complex, depending on cell type, senescence induction method, and autophagy subtype. In Ras‐induced senescence, enhanced autophagy activity contributes to SASP secretion (Narita et al. 2011). In senescent cells, autophagy leads to a decrease in SIRT1 levels (Xu et al. 2020). Furthermore, autophagy activity is enhanced in aging lens epithelial cells (LECs) (Huang et al. 2022). Some inducers of senescence, such as the oncogene Ras and DNA damage, also activate autophagy (Eliopoulos et al. 2016; Narita et al. 2009). Autophagy exerts divergent effects on senescence: it demonstrates anti‐aging properties in some cell types (e.g., neurons, satellite cells, melanocytes, fibroblasts; García‐Prat et al. 2016; Moreno‐Blas et al. 2019; Ni et al. 2016), where autophagy blockade accelerates the senescence process. Conversely, in melanoma models, autophagy promotes senescence to suppress tumorigenesis (Liu et al. 2014). Moreover, autophagy activity is enhanced in RAS‐induced cells, and the LC3‐Lamin B1 interaction plays an important role in enhancing cellular senescence (Dou et al. 2015). During oxidative stress‐induced fibroblast senescence, autophagic flux decreases (Tai et al. 2017), whereas in replicative and oncogene‐induced fibroblast senescence, it increases (Gamerdinger et al. 2009; Young et al. 2009). Different types of autophagy also play distinct roles in cellular senescence. Kang et al. (2015) found that the transcription factor GATA4, a positive regulator of the SASP, is degraded through selective autophagy mediated by p62. Inhibiting selective autophagy in senescent cells increases GATA4 levels and promotes SASP, whereas inhibiting global autophagy alleviates the senescent phenotype. These observations suggest that global autophagy and selective autophagy have opposing effects, with global autophagy promoting and selective autophagy alleviating senescence.

Our data show elevated autophagy‐related proteins in senescent versus young cells (Figure 3A), yet electron microscopy reveals no difference in autophagosome numbers (Figure 3C). Notably, ASNase treatment significantly increases both autophagy‐related proteins and autophagosomes in young and senescent cells (Figure 3B,C), likely due to cellular stress or amino acid deprivation‐induced autophagy activation. This ASNase‐triggered autophagy contributes to suboptimal clinical efficacy of ASNase monotherapy. Autophagy inhibition enhances ASNase sensitivity in ALL (Takahashi et al. 2017) and lung adenocarcinoma (Zhang et al. 2016) by mitigating ASNase‐induced ROS; our results indicate it also further restricts Asn availability since supplementing Asn partially reverses the senolytic effect of “autophagy inhibitor + ASNase”, confirming Asn restriction's essential role in eliminating senescent cells. Similarly, Hinze et al. (2019) demonstrate synthetic lethality between ASNase and proteasome inhibition in acute leukemia—proteasome blockade limits Asn availability by reducing protein degradation. These findings suggest autophagy inhibition could enhance efficacy against clinically ASNase‐insensitive cell lines.

Side effects and resistance represent two significant challenges in the clinical application of ASNase. Currently, clinically used ASNase is primarily derived from *Escherichia coli* (EcA II). Due to its origin and production process, it may elicit immune reactions and toxic responses, such as pancreatitis (Brumano et al. 2018). Additionally, due to the action of human proteases and antibodies, ASNase demonstrates low stability in serum and is rapidly cleared from the plasma. Current solutions primarily involve genetic engineering to modify the enzyme structure, optimizing the purification process, and employing nano‐encapsulation techniques to reduce ASNase's immunogenicity and toxicity, thereby enhancing its stability (Díaz‐Barriga et al. 2021). Resistance frequently occurs in ASNase treatment of acute lymphoblastic leukemia (ALL), primarily because the depletion of Asn activates the amino acid response (AAR), leading to increased ASNS expression via the GCN2‐eIF2‐ATF4 axis (Van Trimpont et al. 2022). Our data reveal a paradoxical state in senescent cells: while upstream integrated stress response markers (phospho‐GCN2 and phospho‐eIF2α) show partial activation, ATF4 expression is significantly reduced at both transcriptional and translational levels—consistent with prior reports (Payea et al. 2024). This implies that resistance observed in tumor cells may not manifest in senescent cells.

HCQ is widely used to treat malaria, autoimmune diseases, and as an adjunct in cancer therapy, with retinal damage being its primary side effect (Bansal et al. 2021). HCQ has a prolonged half‐life (approximately 1 month) and requires about 6 months to be fully eliminated from the body. Side effects are typically observed in long‐term users, with an overall prevalence of 7.5% in patients taking HCQ for more than 5 years, increasing to nearly 20% after 20 years of treatment (Melles and Marmor 2014). Early ocular discomfort can be fully reversed upon discontinuation of treatment. In our regimen, a 3‐day consecutive administration prevents senescent cells from acquiring Asn, thereby facilitating their clearance. This treatment regimen is short‐term, mitigating serious side effects from long‐term drug accumulation and avoiding increased cancer risk by inhibiting physiological senescence. Following treatment with HCQ and ASNase, mice did not exhibit systemic toxicity or significant discomfort. Blood routine and biochemical analyses indicated normal systemic parameters. Nonetheless, further experimental data are required to validate the efficacy and side effects of HCQ and ASNase.

This study identifies ASNS and Asn metabolic abnormality in SNCs, indicating that targeting these abnormalities may offer a novel approach to clear SNCs. This offers new insights into strategies to delay aging and treat age‐related diseases.

## 4 Methods

### 4.1 Ethics Statement

All animal experiments were approved by the Biomedical Ethics Committee of Peking University (approval no. LA2022296). All animal housing and experiments were conducted in strict accordance with the institutional guidelines for care and use of laboratory animals.

### 4.2 Antibodies and Reagents

The antibodies used in this study are listed: ASNS (Proteintech, 14681‐1‐AP), P21 (Abcam, ab109520), P16 (Abcam, ab108349), P16 (Abcam, ab54210), P53 (Santa Cruz, sc126), ATF4 (ABclonal, A18687), LC3B (ABclonal, A5618), BECN1 (ABclonal, A11761), ATG7 (ABclonal, A19604), P62 (Proteintech,18,420–1‐AP), PSMB5 (ABclonal, A1975), PSMB6 (ABclonal, A4053), PSMB7(ABclonal, A14771), p‐GCN2(Thr899) (ABclonal, AP1428), GCN2(ABclonal, A2307), p‐PERK(T982) (ABclonal, AP1501), PERK (ABclonal, A21255), p‐eIF2α (S51) (ABclonal, AP0745), eIF2α (ABclonal, A21221), Bcl‐XL (Proteintech,26967‐1‐AP), Bax (Proteintech,60267–1‐lg), Caspase3(abcam, Ab32351), C‐Caspase3(CST,9664S), and C‐Caspase9(CST, 9505P).

The other compounds used in this study are listed along with their manufacturers' details: Gemcitabine (Selleck, S1714), Bleomycin (TargetMol, T6116), Dasatinib (Aladdin, D125110), Quercetin (Sigma, Q4951), L‐ASNase (MCE, HY‐P1923), Hydroxychloroquine (TargetMol, T9287), L‐Asn (Sigma, A0884), L‐Aspartic acid (Sigma, A6558), and glutamic acid (Sigma, G3640), Lys05 (TargetMol, T3437).

### 4.3 Cell Culture, Plasmid Construction, and Transfection

All cells were cultured in a 5% CO_2_ humidified incubator at 37°C. The cells, namely, 293T, 2BS, MEFs, ARPE‐19, and human pancreatic ductal adenocarcinoma cell lines, viz. PANC‐1 and Mia Paca2, were routinely maintained in standard Dulbecco's modified Eagle's medium (DMEM) with 10% fetal bovine serum (FBS) and 1% penicillin–streptomycin. The primary HUVECs were grown in endothelial cell medium supplemented with 5% FBS, 1% endothelial cell growth supplement, and 1% penicillin–streptomycin. MEFs were isolated and cultured as described (Tan and Lei 2019).

Lentiviral vectors for ASNS, BECN1, and ATG7 shRNA were constructed based on the pLKO.1 plasmid. The shRNA sequences for humans are listed.

shScramble‐F:5′‐CCGGCAACAAGATGAAGAGCACCAACTCGAGTTGGTGCTCTTCATCTTGTTGTTTTT‐3′

shScramble‐R: 5′‐aattAAAAACAACAAGATGAAGAGCACCAACTCGAGTTGGTGCTCTTCATCTTGTTG‐3′ shASNS‐F: 5′‐CCGGGCTCTGTTACAATGGTGAAATCTCGAGATTTCACCATTGTAACAGAGCTTTTTG‐3′, shASNS‐R: 5′‐aattCAAAAAGCTCTGTTACAATGGTGAAATCTCGAGATTTCACCATTGTAACAGAGC‐3′; shBECN1‐F: 5′‐CCGGGGTCTAAGACGTCCAACAACACTCGAGTGTTGTTGGACGTCTTAGACCTTTTTG‐3′, shBECN1‐R: 5′‐aattCAAAAAGGTCTAAGACGTCCAACAACACTCGAGTGTTGTTGGACGTCTTAGACC‐3′; and ShATG7‐F: 5′‐CCGGGCTGGTTTCCTTGCTTAAACACTCGAGTGTTTAAGCAAGGAAACCAGCTTTTTG‐3′, ShATG7‐R: 5′‐aattCAAAAAGCTGGTTTCCTTGCTTAAACACTCGAGTGTTTAAGCAAGGAAACCAGC‐3′.

Human ASNS coding sequences with flag‐tags and human ATF4 coding sequences, amplified from a cDNA library, were cloned into a PCDH vector. Sequences for cloning are listed. PCDH‐ASNS‐F:5′‐GTCAGATCCGCTAGCCACCATGTGTGGCATTTGGGCGCT‐3′; PCDH‐ASNS‐R:5′‐GTCACGCGTGAATTCAGCTTTGACAGCTGACTTGT‐3′ PCDH‐ATF4‐F: 5′‐GATTCTAGAGCTAGCCACCATGACCGAAATGAGCTTCCTGAG‐3′; PCDH‐ATF4‐R:5′‐CGCGGCCGCGGATCCCTAGGGGACCCTTTTCTTCC‐3′. Transfection was performed following Vigofect (Vigorous Biotechnology) instructions.

### 4.4 Cellular Senescence Induction

Bleomycin‐induced premature senescence was conducted with 60%–70% confluent cells treated with 50 μg/mL bleomycin. After 24 h, the complete culture medium was added, and the culture medium was changed every 2 d for 7 d.

IR‐induced premature senescence was conducted with 60%–70% confluent cells treated with 10 Gy IR. After 24 h, the complete culture medium was added, and the culture medium was changed every 2 d for 7 d.

Replicative senescence was conducted with 2BS cells that have been passaged 50–60 times, HUVECs that have been passaged 10–13 times, and MEFs that have been passaged 8–10 times.

The following were considered young cells: 2BS cells within 25 passages, HUVECs within 5 passages, and MEFs within 4 passages.

### 4.5 Animals

All animal experiments were performed according to the animal protection guidelines of Peking University Health Science Center, China. All animals were maintained in a specific pathogen‐free facility with a 12 light/12 dark cycle and free access to food and water. C57BL/6J, SAMP6, and ApoE^−/−^ mice were randomized into different groups.

### 4.6 Physical Function Measurements

All functional assays were conducted at least 5 d after the last drug dose was administered. Mice were trained at least two times on d 1 and 2 and tested on d 3, 4, and 5. Results presented are the average of three trials.

For rotarod test, mice were placed on the rotarod device (Unibiolab) with the following settings: an initial speed of 5 rpm, an acceleration rate of 0.1 rpm/s, a maximum speed of 30 rpm, and a cutoff time of 600 s. The device automatically recorded the speed and time at which the mice fell off the rod.

For grid test, mice were placed on the grip strength meter (Unibiolab), with all four limbs grasping the spring of the device. The mice were then gently pulled parallel to the device by their tails. The maximum and average grip strength (N) were recorded for five trials.

For open field test, after acclimating to the open field apparatus (Unibiolab) for 30 min, the mice were placed in a 40 cm square box. A camera recorded their activity for 10 min, capturing movement trajectories, speed, and other behaviors. The environment was kept bright and quiet, and the apparatus was cleaned to remove any odors.

### 4.7 Blood Analysis

For routine blood examination, 50 μL fresh blood was collected from each mouse and mixed with EDTA immediately. The blood samples were analyzed by an automatic blood cell analyzer (Mindray, BC‐2800vet).

For serum biochemical analysis, blood samples were collected, clotted for 45 min at 37°C and then overnight at 4°C, and later centrifuged (1000×*g*, 10 min) to obtain serum. Serum aliquots (200 μL) were analyzed for ALT, aspartate transaminase (AST), carbamide (UREA), and creatinine (CREA) by using a chemical analyzer (Mindray, Chemray 800).

### 4.8 Histological Analysis

Tissues were fixed overnight in 4% paraformaldehyde, embedded in paraffin, and cut into 5 μm sections. Sections were subjected to hematoxylin and eosin staining and Masson's trichrome staining for collagen detection. The collagen‐positive area was quantified with ImageJ. Immunohistochemical and immunofluorescence staining was performed following standard protocols. The following primary antibodies were used: ASNS (Proteintech, 14681‐1‐AP), P16 (Abcam, ab54210), Lamin B1(afantibody, AFRM0038).

### 4.9 SA‐Beta‐Gal Staining

SA‐beta‐gal activity was performed as described (Shlush and Selig 2013). Briefly, freshly frozen tissue sections or adherent cells were washed twice with cold PBS and fixed with 4% paraformaldehyde for 5 min at room temperature. After two cold PBS washes, the samples were incubated with freshly prepared SABG staining solution (containing 1 mM MgCl_2_, 1 mg/mL X‐gal, 5 mM potassium ferricyanide, and 5 mM potassium ferrocyanide) overnight at 37°C. Staining was ended by washing three times with cold PBS.

### 4.10 Oil Red O Staining

Atherosclerotic plaque was detected with oil red O staining. Briefly, whole aortas were separated and fixed in 4% paraformaldehyde for 12 h at room temperature. Then, the fixed aortas were incubated in 0.5% oil red O solution prepared in 60% isopropyl alcohol for 15 min at 37°C. Subsequently, the samples were washed in 60% isopropyl alcohol solution for 1 min and washed with double distilled water. The oil red O‐stained aortas were dissected and pinned on foam flat. After the specimens were photographed, quantification of the plaque area was done with ImageJ.

### 4.11 CCK‐8 Assay

Cells were seeded into 96‐well plates (3000 cells/well) with 100 μL medium containing 10% FBS. Next, the cells were treated with 100 μL DMEM and CCK‐8 (Dojindo, Japan) solution (90:10) at 37°C for 1 h; absorbance was measured at 450 nm using a 96‐well plate reader.

### 4.12 ELISA Analysis

Mouse whole blood samples were stewed at 37°C for 1 h and overnight at 4°C. Then, the samples were centrifuged at 3000 rpm for 10 min at 4°C. The supernatant is serum. IL1β and IL6 in serum were measured by mouse interleukin 1 beta (IL1β) ELISA kit (Servicebio, GEM0002) and mouse interleukin 6 (IL6) ELISA kit (Servicebio, GEM0001), respectively. GSH‐PX, T‐SOD, and MDA were measured by glutathione peroxidase (GSH‐PX) (Njjcbio, A005), total superoxide dismutase (T‐SOD) (Njjcbio, A001‐1), and MDA (Njjcbio, A003‐1) assay kits, respectively, according to the manufacturer's protocols.

### 4.13 Apoptosis Detection

Cells were digested into single‐cell suspension by incubation with 0.25% trypsin at 37°C for 3 min. Cells were washed three times with PBS and stained with APC annexin V using the annexin V‐APC apoptosis detection kit (Elabscience, E‐CK‐A217) and propidium iodide according to the manufacturer's protocol. Cell apoptosis was detected by flow cytometry on an Accuri c6 Plus (BD). Data were analyzed using FlowJo software. Apoptotic cells were annexin V‐positive cells.

### 4.14 RT‐qPCR

Total RNA was extracted from cells with TransZol reagent (TransGen Biotech, ET101‐01‐V2) and reverse transcribed into cDNA using TransScriptII all‐in‐one first strand cDNA synthesis supermix (TransGen Biotech, AH341‐01). The cDNA was quantitated by RT‐qPCR with PerfectStart green qPCR supermix (TransGen Biotech, AQ602‐01). Data were analyzed using the 2^(−ΔΔCt)^ method. GAPDH and ACTB served as endogenous normalization controls for mouse and human samples, respectively. The primers used are mentioned as follows.

Mouse primers:

GAPDH‐F: 5′‐TCACTGCCACCCAGAAGAC‐3′, GAPDH‐R: 5′‐TGTAGGCCATGAGGTCCAC‐3′; Il1b‐F: 5′‐TTGACAGTGATGAGAATGACC‐3′, Il1b‐R: 5′‐GCAGGTTATCATCATCATCC‐3′; Il6‐F: 5′‐ACCACTCCCAACAGACCTGTCTATACC‐3′, Il6‐R: 5′‐CTCCTTCTGTGACTCCAGCTTATCTGTTAG‐3′; MMP3‐F: 5′‐TCCTGATGTTGGTGGCTTCAG‐3′, MMP3‐R: 5′‐TGTCTTGGCAAATCCGGTGTA‐3′; Il8‐F: 5′‐TCGAGACCATTTACTGCAACAG‐3′, Il8‐R: 5′‐CATTGCCGGTGGAAATTCCTT‐3′; and ASNS‐F: 5′‐GCAGTGTCTGAGTGCGATGAA‐3′, ASNS‐R: 5´‐TCTTATCGGCTGCATTCCAAAC‐3′.

Human primers:

GAPDH‐F: 5′‐ACAACAGCCTCAAGATCATCAGCAAT‐3′, GAPDH‐R: 5′‐GTCCTTCCACGATACCAAAGTTGTCA‐3′; ASNS‐F: 5′‐GGAAGACAGCCCCGATTTACT‐3′, ASNS‐R: 5′‐AGCACGAACTGTTGTAATGTCA‐3′; ATG7‐F: 5′‐CAGTTTGCCCCTTTTAGTAGTGC‐3′, ATG7‐R: 5′‐CCAGCCGATACTCGTTCAGC‐3′; BECN1‐F: 5′‐GGTGTCTCTCGCAGATTCATC‐3′, BECN1‐R: 5′‐TCAGTCTTCGGCTGAGGTTCT‐3′; IL1B‐F: 5′‐ATGATGGCTTATTACAGTGGCAA‐3′, IL1B‐R: 5′‐GTCGGAGATTCGTAGCTGGA‐3′; IL6‐F: 5′‐ACTCACCTCTTCAGAACGAATTG‐3′, IL6‐R: 5′‐CCATCTTTGGAAGGTTCAGGTTG‐3′; IL8‐F: 5′‐ACTGAGAGTGATTGAGAGTGGAC‐3′, IL8‐R: 5′‐AACCCTCTGCACCCAGTTTTC‐3′; CXCL1‐F: 5′‐TTGCCTCAATCCTGCATCCC‐3′, CXCL1‐R: 5′‐AGTTGGATTTGTCACTGTTCAGC‐3′; MMP3‐F: 5′‐CGGTTCCGCCTGTCTCAAG‐3′, MMP3‐R: 5′‐CGCCAAAAGTGCCTGTCTT‐3′; ATF4‐F: 5′‐CCCTTCACCTTCTTACAACCTC‐3′, ATF4‐R: 5′‐TGCCCAGCTCTAAACTAAAGGA‐3′.

### 4.15 Western Blot

Cells were lysed by using RIPA buffer (Beyotime, P0013B) on ice for 10–20 min. Protein samples were quantified using BCA protein assay kit (Beyotime, P0010), boiled in 5× loading buffer. The samples containing 20–50 μg total protein were subjected to 6%–12% SDS‐PAGE and transferred to a 0.45 μm polyvinylidene fluoride membrane (Millipore). The membrane was blocked with 5% BSA (Biorigin, BN20800) and incubated overnight with indicated primary antibodies in TBS supplemented with 0.1% Tween 20 at 4°C. The membrane was then incubated with horseradish peroxidase (HRP)‐labeled IgG secondary antibodies at room temperature for 1 h, and the membrane was developed with chemiluminescent HRP substrate (Millipore, P90720) on a ChemiDoc imaging system (BioRad, USA). The band intensity was quantified with ImageJ software (https://imagej.en.softonic.com/).

### 4.16 LC–MS/MS

Liquid chromatography–tandem mass spectrometry (LC–MS/MS) was employed for quantitative analysis of asparagine (Asn) and glutamic acid (Glu). For fluid samples, metabolites in 100 μL mouse serum or 100 μL supernatants collected from the culture medium were extracted by precooled 100% acetonitrile in a ratio of 1:5. For cell samples, adhesive cells were harvested and washed three times with normal saline; the viable cell count was recorded prior to metabolite extraction. Metabolites from cells cultured in a 6 mm dish were extracted by 500 μL precooled 100% acetonitrile. Then, the mixtures were vortexed for 5 min and centrifuged at 14,000 × g for 20 min at 4°C. Metabolites in these supernatants were collected and analyzed using a triple quadrupole LC–MS/MS system (Agilent, 1290/6460).

### 4.17 Micro‐CT

Following cervical dislocation euthanasia, left femurs were dissected free of connective tissues and fixed overnight in 4% paraformaldehyde at 4°C. After PBS rinses (3×), specimens were stored in 75% ethanol prior to micro‐CT scanning (SkyScan 1276, Bruker MicroCT) using standardized parameters: 50 kV source voltage, 200 μA current, and 6.054735 μm isotropic voxel size. Three‐dimensional reconstructions were generated via NRecon software (v1.7.4.2) with trabecular morphometric analysis—including bone mineral density (BMD) calibrated against hydroxyapatite phantoms and microarchitectural parameters (BV/TV, Tb.Th, Tb.Sp, Tb.N)—performed on distal femoral metaphyseal regions using CTAn software (v1.20.8.0).

### 4.18 Transmission Electron Microscopy

Cells sample preparation commenced with primary fixation of cell pellets using 2.5% glutaraldehyde in 0.1 M phosphate buffer (pH 7.4) for 2 h at 4°C. Following triple PBS washes, secondary fixation was performed with 1% osmium tetroxide for 1.5 h prior to en bloc staining with 0.5% uranyl acetate. Specimens were dehydrated through an ethanol series (30%–100%) and acetone, then embedded in Epon‐Araldite resin via graded infiltration before polymerization at 60°C for 48 h. Ultrathin sections (70 nm nominal thickness) were cut using a Leica EM UC7 ultramicrotome equipped with a 45° diamond knife (Diatome), mounted on 200‐mesh copper grids, and post‐stained with 2% uranyl acetate and Reynolds' lead citrate. Imaging was conducted on a JEOL JEM‐1400 transmission electron microscope operated at 80 kV.

### 4.19 Statistical Analysis

All statistical analyses were performed using SPSS 20 (IBM) and GraphPad Prism. Data distribution was assumed to be normal, but this was not tested. Data are presented as mean ± SEM. Unpaired two‐tailed *t* tests were used to estimate statistically significant differences between two groups. One‐way ANOVA test with Holm–Sidak multiple test correction was used for multiple comparisons. Two‐way ANOVA test with Tukey's post hoc comparison was used for CCK8 assay. *p < 0.05, **p < 0.01, ***p < 0.001, and ns, not significant.

## Author Contributions

W.Y. and Z.M. conceived and supervised this study. W.Y., Z.M., and Y.Y. were responsible for all aspects of study design. Z.H., X.L., X.Z., K.C., H.D., M.W., J.W., and Z.L. conducted the experiments, interpreted the results, and performed the statistical analysis. W.Y., Z.M., Z.H., and X.L. wrote the manuscript. Z.H., X.L., X.Z., K.C., H.D., M.W., J.W., and Z.L. verified the underlying data. All authors critically reviewed the manuscript and approved the final version.

## Conflicts of Interest

The authors declare no conflicts of interest.

## Supporting information


**Figure S1:** Aging Atlas reveals decreased ASNS expression in senescent cells.
**Figure S2:** Decreased Asn levels in senescent cells.
**Figure S3:** Reduced ATF4 expression in senescent cells impairs ASNS transcription.
**Figure S4:** ASNS influences cellular senescence through Asn.
**Figure S5:** ASNase induces apoptosis in senescent cells.
**Figure S6:** ASNase and autophagy inhibition promote apoptosis in senescent cells by limiting asparagine availability.
**Figure S7:** Autophagy inhibitor HCQ induces apoptosis in senescent cells.
**Figure S8:** AH treatment triggers activation of apoptotic pathway in senescent cells.
**Figure S9:** ASNase and autophagy inhibitior Lys05 promote apoptosis in senescent cells.
**Figure S10:** AH kills senescent HUVECs.
**Figure S11:** AH kills various types of senescent cells.
**Figure S12:** AH eliminates TIS tumor cells by limiting asparagine availability.
**Figure S13:** AH deletes senescent cells of aged mice.
**Figure S14:** AH deletes senescent cells and improves physical function of aged mice.
**Figure S15:** AH delays the progression of senile osteoporosis.
**Figure S16:** AH delays the progression of atherosclerosis.
**Figure S17:** AH treatment on murine mesenchymal stem cells.
**Figure S18:** The treatment of AH showed no obvious systemic toxicity.
**Figure S19:** Preservation of hepato‐renal morphology following AH administration.
**Figure S20:** A model of cellular asparagine source and AH treatment kills senescent cells effectively and selectively.

## Data Availability

Data sharing is not applicable to this article as no new data were created or analyzed in this study.
