# Supplementary material for: Asparaginase and Autophagy Inhibitors Effectively Remove Senescent Cells by Synergistically Limiting Asparagine Supply
Source: Aging Cell. 2025 Sep 4;24(10):e70203. doi: 10.1111/acel.70203 (PMC12507409; doi:10.1111/acel.70203)
Supplement: Supplementary file 1 — Figure S1: Aging Atlas reveals decreased ASNS expression in senescent cells. Figure S2: Decreased Asn levels in senescent cells. Figure S3: Reduced ATF4 expression in senescent cells impairs ASNS transcription. Figure S4: ASNS influences cellular senescence through Asn. Figure S5: ASNase induces apoptosis in senescent cells. Figure S6: ASNase and autophagy inhibition promote apoptosis in senescent cells by limiting asparagine availability. Figure S7: Autophagy inhibitor HCQ induces apoptosis in senescent cells. Figure S8: AH treatment triggers activation of apoptotic pathway in senescent cells. Figure S9: ASNase and autophagy inhibitior Lys05 promote apoptosis in senescent cells. Figure S10: AH kills senescent HUVECs. Figure S11: AH kills various types of senescent cells. Figure S12: AH eliminates TIS tumor cells by limiting asparagine availability. Figure S13: AH deletes senescent cells of aged mice. Figure S14: AH deletes senescent cells and improves physical function of aged mice. Figure S15: AH delays the progression of senile osteoporosis. Figure S16: AH delays the progression of atherosclerosis. Figure S17: AH treatment on murine mesenchymal stem cells. Figure S18: The treatment of AH showed no obvious systemic toxicity. Figure S19: Preservation of hepato‐renal morphology following AH administration. Figure S20: A model of cellular asparagine source and AH treatment kills senescent cells effectively and selectively. [file ACEL-24-e70203-s001.docx]

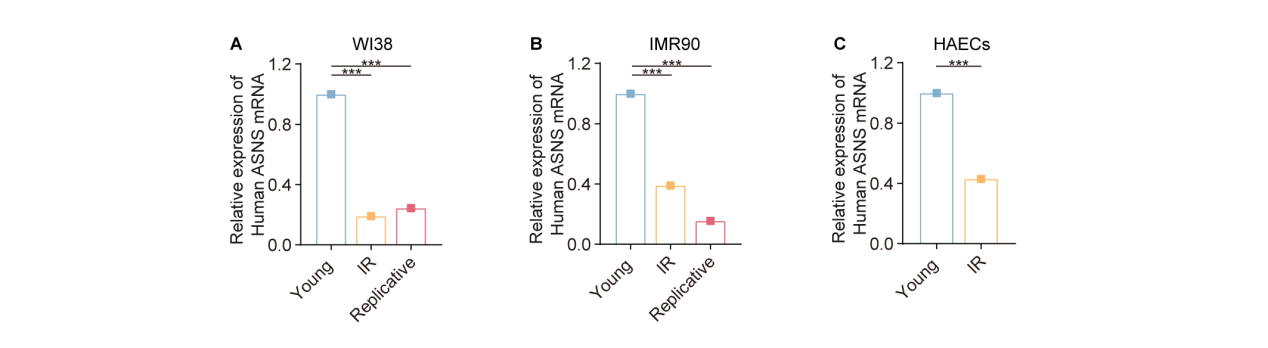


**Fig.1 Aging Atlas reveals decreased ASNS expression in senescent cells.** A-C,Expression data of ASNS mRNA from Aging Atlas(https://ngdc.cncb.ac.cn/aging/index).(A)Human diploid fibroblasts(WI38),(B)Human diploid fibroblasts(IMR90),(C)Human arterial endothelial cell (HAECs).Data are presented as means ± SEM. One-way ANOVA test for (A-C), ****P* < 0.001.


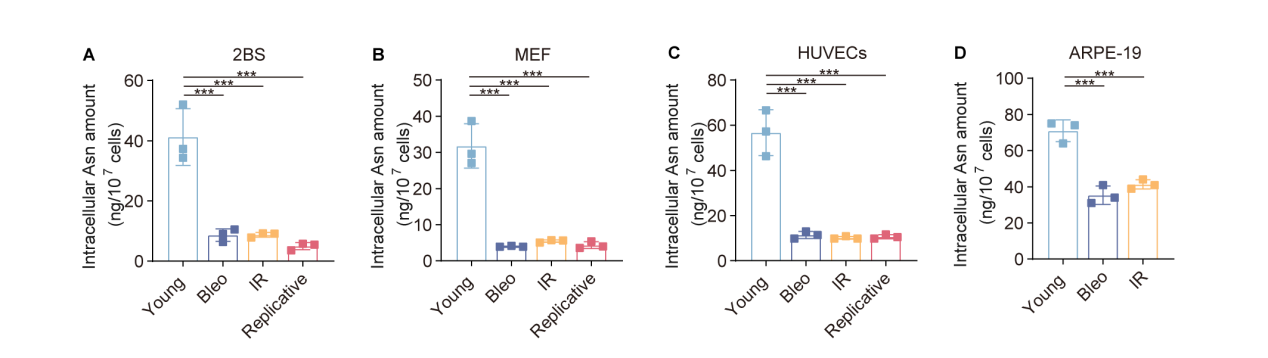


**Fig.2 Decreased Asn levels in senescent cells.**A-D,Detection of intracellular Asn levels by LC-MS/MS.(A)2BS,(B)MEF,(C)HUVECs, (D)ARPE-19.Data are presented as means ± SEM. One-way ANOVA test for (A-D), ****P* < 0.001.


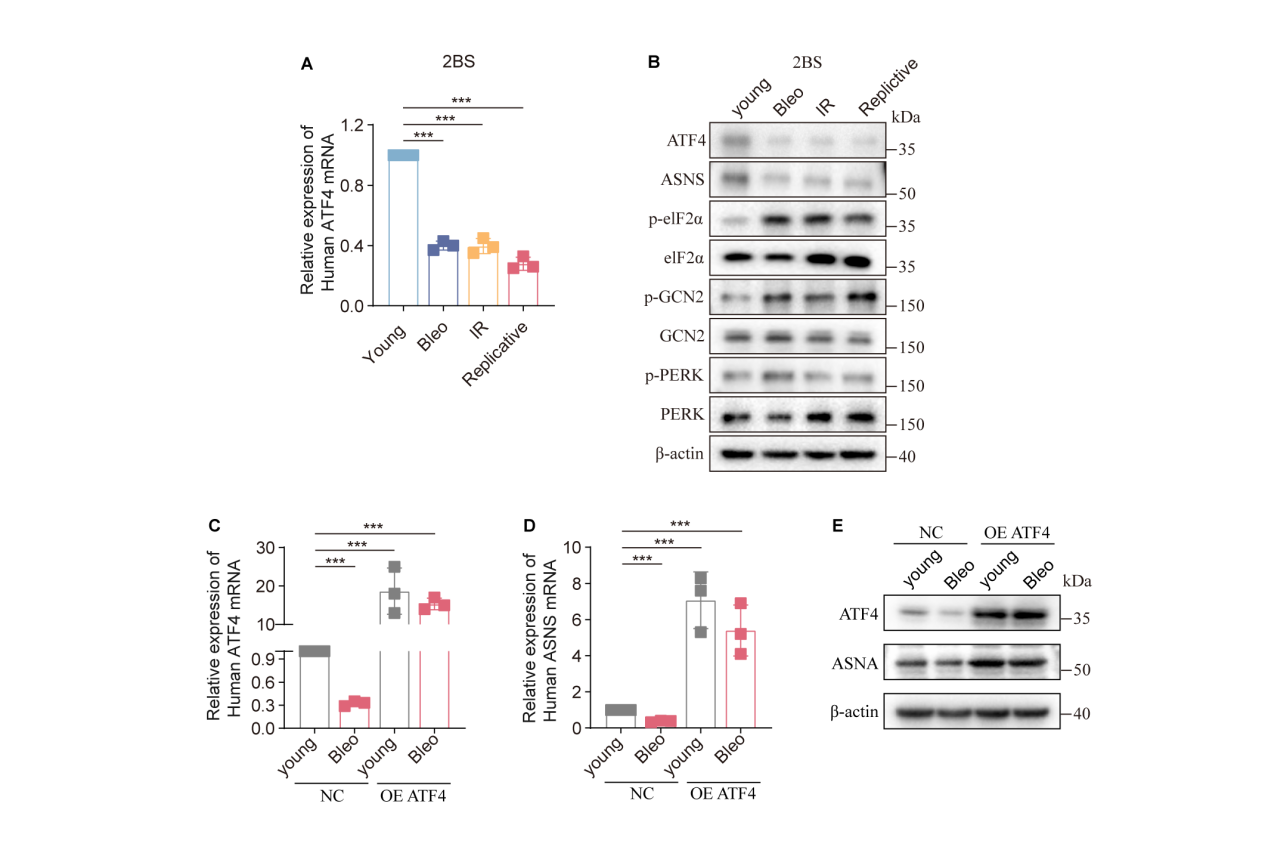


**Fig.3 Reduced ATF4 expression in senescent cells impairs ASNS transcription.**A.Quantification of ATF4 genes by RT-qPCR in bleomycin-induced prematurely senescent 2BS cells(Bleo),γ-ray-induced prematurely senescent 2BS cells(IR) and replicatively senescent 2BS cells(Replicative).B.Quantification of proteins by by western blot in bleomycin-induced prematurely senescent 2BS cells(Bleo),γ-ray-induced prematurely senescent 2BS cells(IR) and replicatively senescent 2BS cells(Replicative).C-E.ATF4 was overexpressed in young and bleomycin-induced senescent 2BS cells via lentiviral transduction.(C,D)Detection of ATF4 and ASNS mRNA by qPCR.(E)Detection of ATF4 and ASNS protein by western blot.Data are presented as means ± SEM. One-way ANOVA test for (A,C,D), ****P* < 0.001.


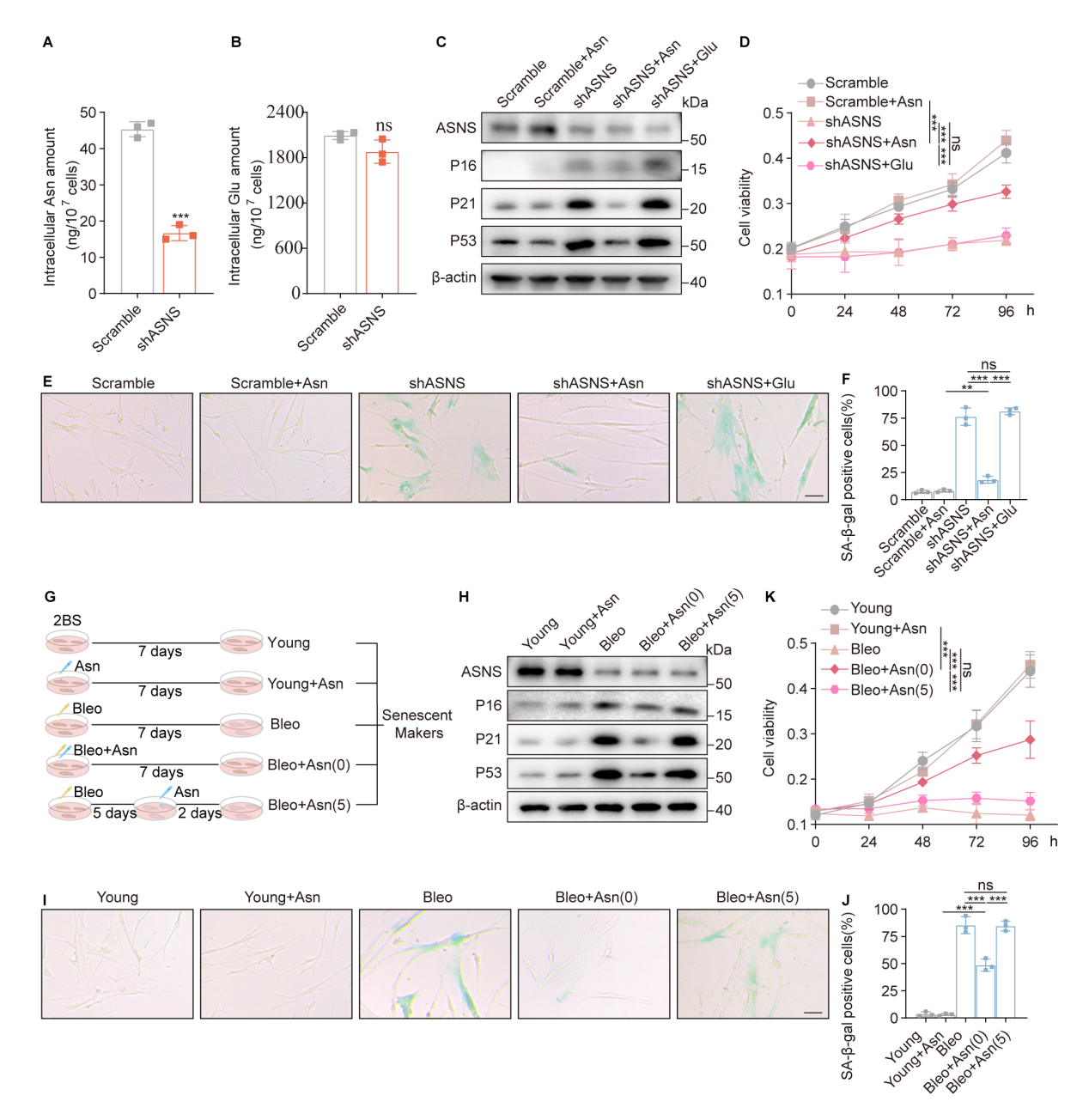


**Fig.4 ASNS influences cellular senescence through Asn.**A,B.Detection of intracellular Asn and Glu levels by LC-MS/MS.C-F.Following infection with lentivirus containing either the pLKO.1-scramble or pLKO.1-shASNS,2BS cells were treat with 0.1 μM Asn or 0.1 μM Glu then cultured for 7 days.(C)Detection ASNS protein and senescence-associated protiens.(D)Quantification of viability of cells(n=3).(E)Representative images (Scale bar, 50 μm)and (F)quantification of SA-β-gal staining(n = 3).G-K,Young 2BS cells were treated with bleomycin(50 μg/ml) to induce senescence while being treated with Asn(0.1 μM). "0" indicates that Asn was added at the start of bleomycin induction, whereas "5" indicates that asn was added 5 days after the start of bleomycin induction. Senescence-associated markers were detected on the seventh day. (G) Experimental design.(H)Detection ASNS protein and senescence-associated protiens. (I)Representative images(Scale bar, 50 μm) and (J)quantification of SA-β-gal staining(n = 3). (K)Quantification of viability of cells(n=3). Data are presented as means ± SEM. Unpaired two-tailed *t*-test for (A,B),One-way ANOVA test for (F,J),two-way ANOVA test for (D,K), ***P* < 0.01, ****P* < 0.001, ns = not significant.


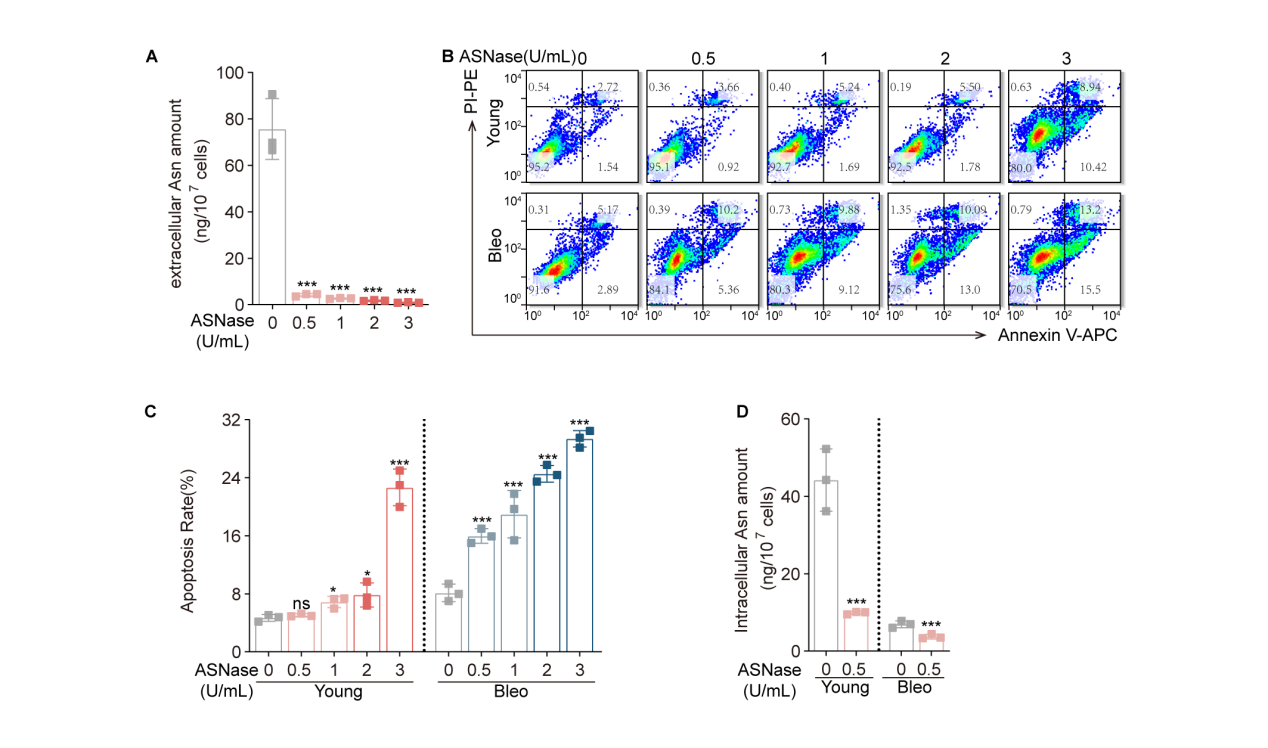


**Fig.5 ASNase induces apoptosis in senescent cells.** A. Detection of extracellular Asn levels in cell supernatant treated with ASNase (0-3 U/mL) by LC-MS/MS (n = 3). B-D. Young and senescent 2BS cells treated with ASNase (0-3 U/mL, 3 days, n = 3). (B) Detection of cell apoptosis by flow cytometry, and (C) Analysis of apoptosis rate,(D) Detection of intracellular Asn levels.Data are presented as means ± SEM. one-way ANOVA test for (A,C,D), **P* < 0.05, ***P* < 0.01, ****P* < 0.001, ns = not significant.

**
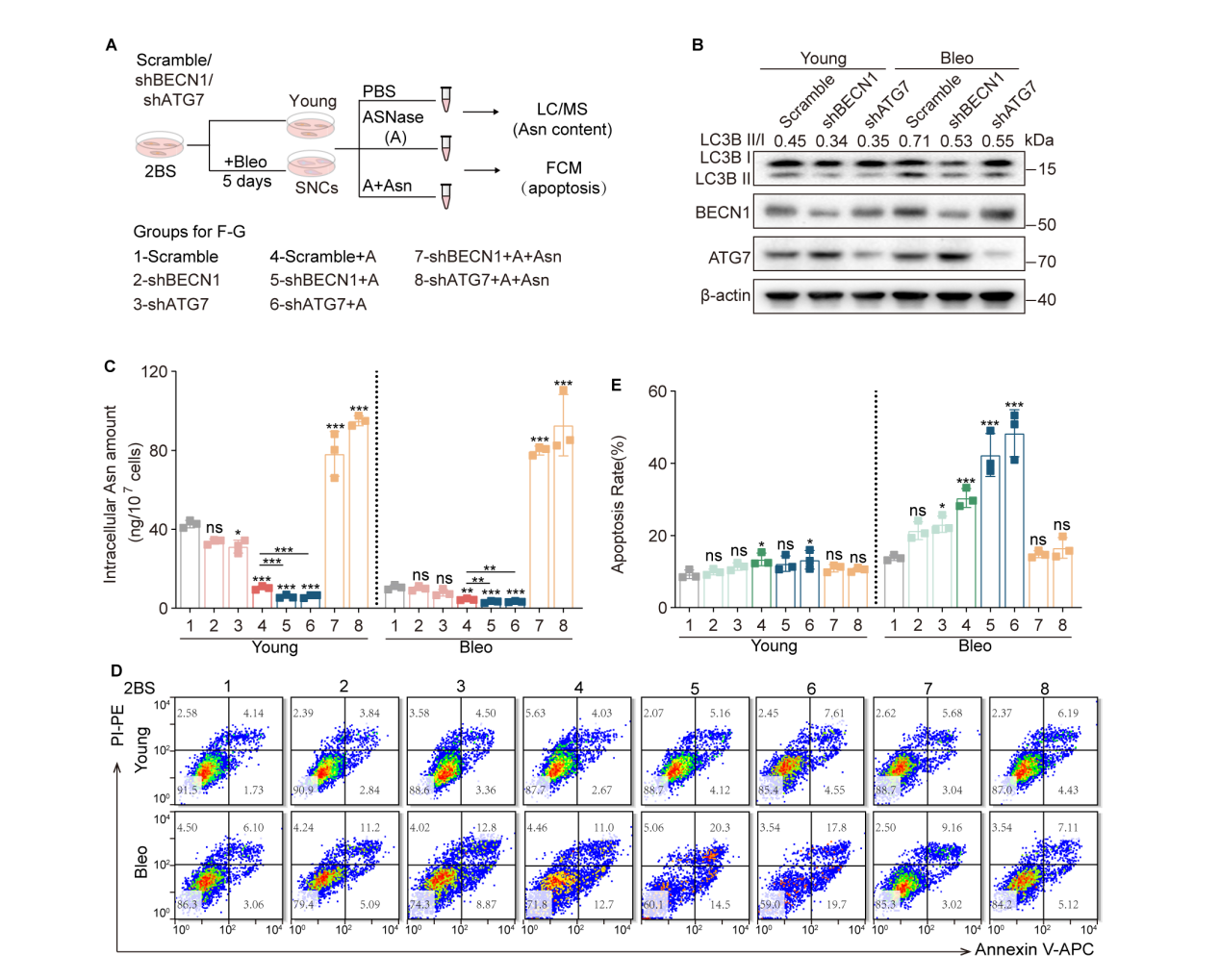

Fig.6 ASNase and autophagy inhibition promote apoptosis in senescent cells by limiting asparagine availability.**

A-E,Young and senescent cells were subjected to the following treatments: "Scramble" indicates cells infected with lentivirus carrying a nonsense shRNA sequence, "shBECN1" indicates cells infected with lentivirus carrying the shBECN1 sequence, "shATG7" indicates cells infected with lentivirus carrying the shATG7 sequence. "+A" indicates treatment with ASNase (0.5 U/mL, 3 days), and "+Asn" indicates treatment with Asn (200 μM) with replenishment every 12 hours after ASNase treatment.(A) Experimental design for cells treated with ASNase and autophagy inhibition. (B)Detection of the knockdown efficiency of BECN1 and ATG7 in young and senescent 2BS cells by Western blot. (C)Detection of intracellular Asn levels by LC-MS/MS, (D)Detection of cell apoptosis by flow cytometry and (E)analysis of apoptosis rate.Data are presented as means ± SEM. one-way ANOVA test for (C,E), **P* < 0.05, ***P* < 0.01, ****P* < 0.001, ns = not significant.


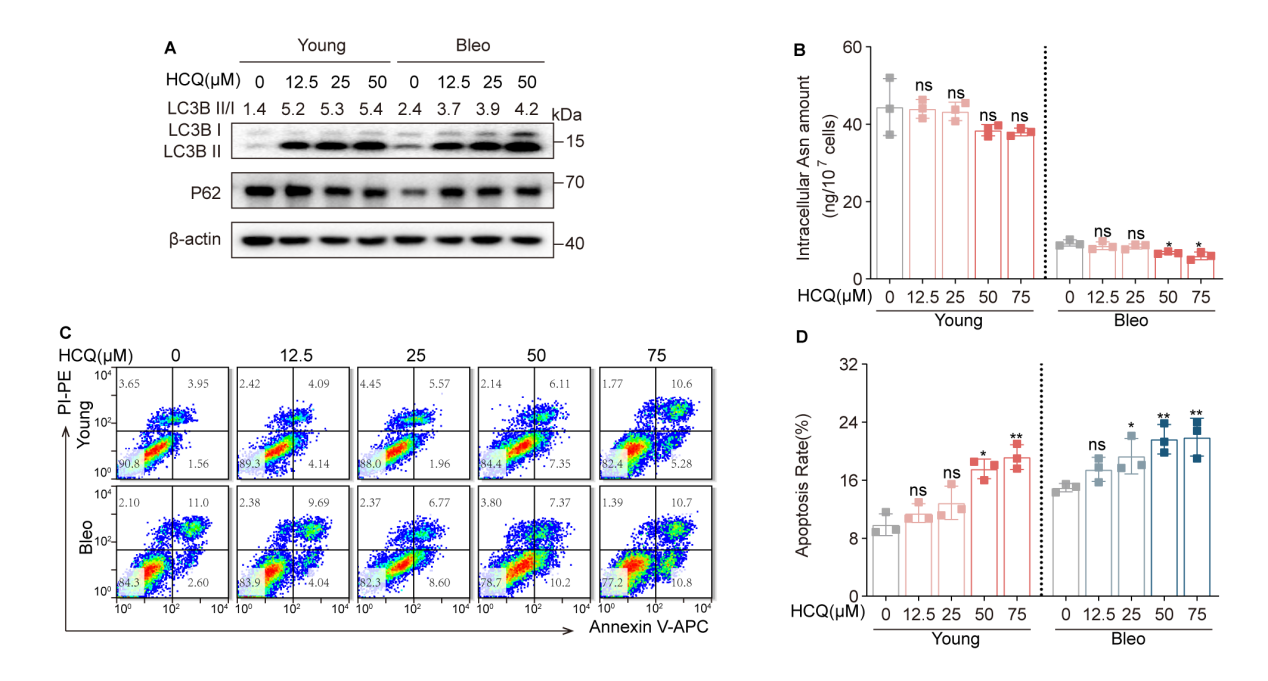


**Fig.7 Autophagy inhibitor HCQ induces apoptosis in senescent cells**

A-D. Young and senescent 2BS cells treated with HCQ (0-75 μM, 3 days, n = 3). (A) Detection of autophagy-related proteins by western blot,(B) Detection of intracellular Asn levels, (C) Detection of cell apoptosis by flow cytometry, (D) Analysis of apoptosis rate. Data are presented as means ± SEM. one-way ANOVA test for (B,D), **P* < 0.05, ***P* < 0.01, ****P* < 0.001, ns = not significant.


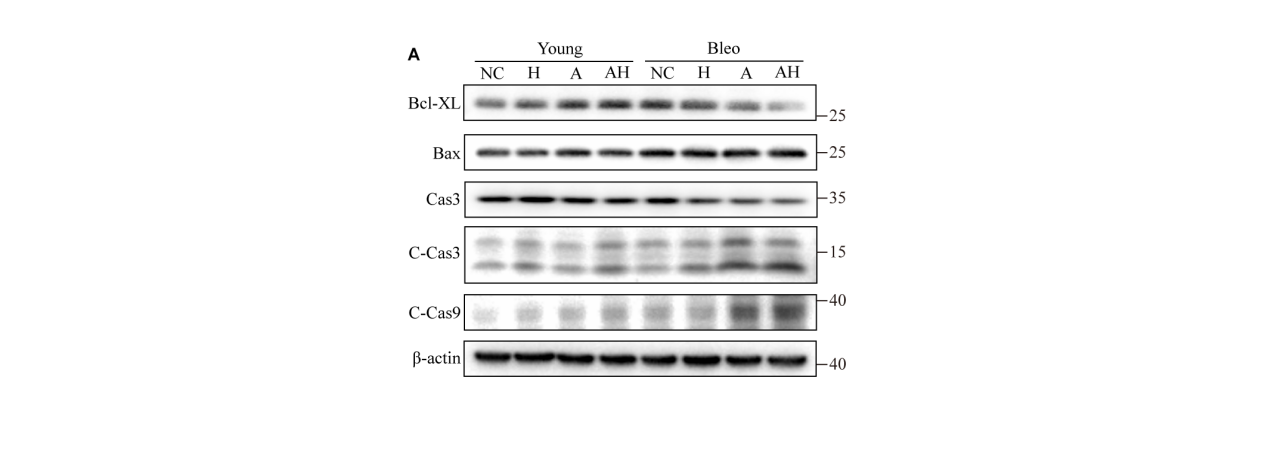


**Fig.8 AH Treatment Triggers Activation of Apoptotic Pathway in Senescent Cells.**A.Young and senescent cells were subjected to the following treatments:"NC" indicates the negative control, "H" indicates cells treated with HCQ (25 μM, 3 days), "A" indicates treatment with ASNase (0.5 U/mL, 3 days), and "AH" indicates simultaneous treatment with both ASNase and HCQ.Western blot analysis of apoptosis-associated proteins.


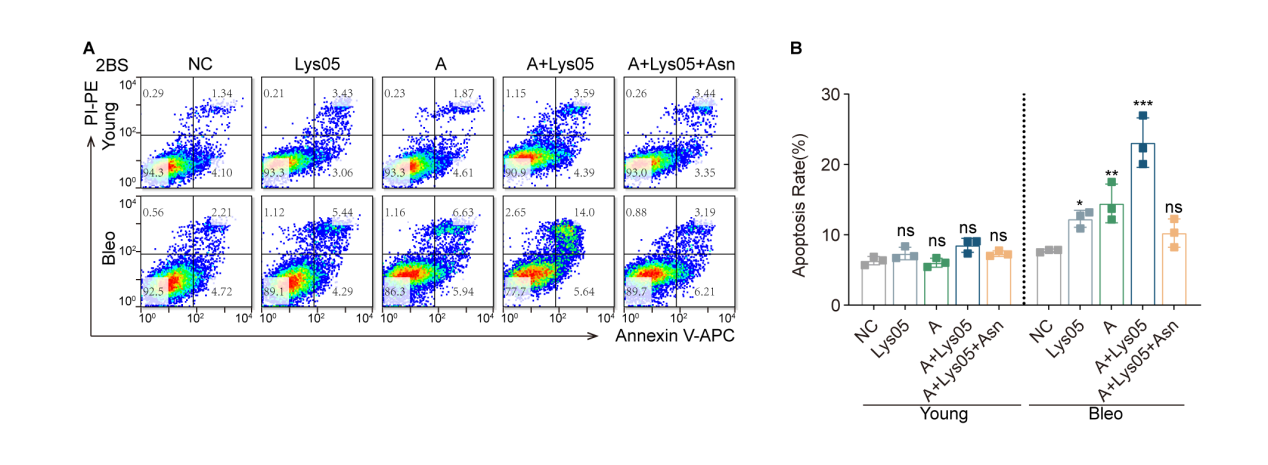


**Fig.9 ASNase and autophagy inhibitior Lys05 promote apoptosis in senescent cells.**Young and senescent cells were subjected to the following treatments:"NC" indicates the negative control, "Lys05" indicates cells treated with Lys05 (5 μM, 3 days), "A" indicates treatment with ASNase (0.5 U/mL, 3 days), "A+Lys05" indicates simultaneous treatment with both ASNase and Lys05, "A+Lys05+Asn" or"Asn" indicates treatment with Asn (200 μM) with replenishment every 12 hours after "A+Lys05" treatment.(A)Detection of cell apoptosis by flow cytometry and (B)analysis of apoptosis rate.Data are presented as means ± SEM. one-way ANOVA test for (B), **P* < 0.05, ***P* < 0.01, ****P* < 0.001, ns = not significant.


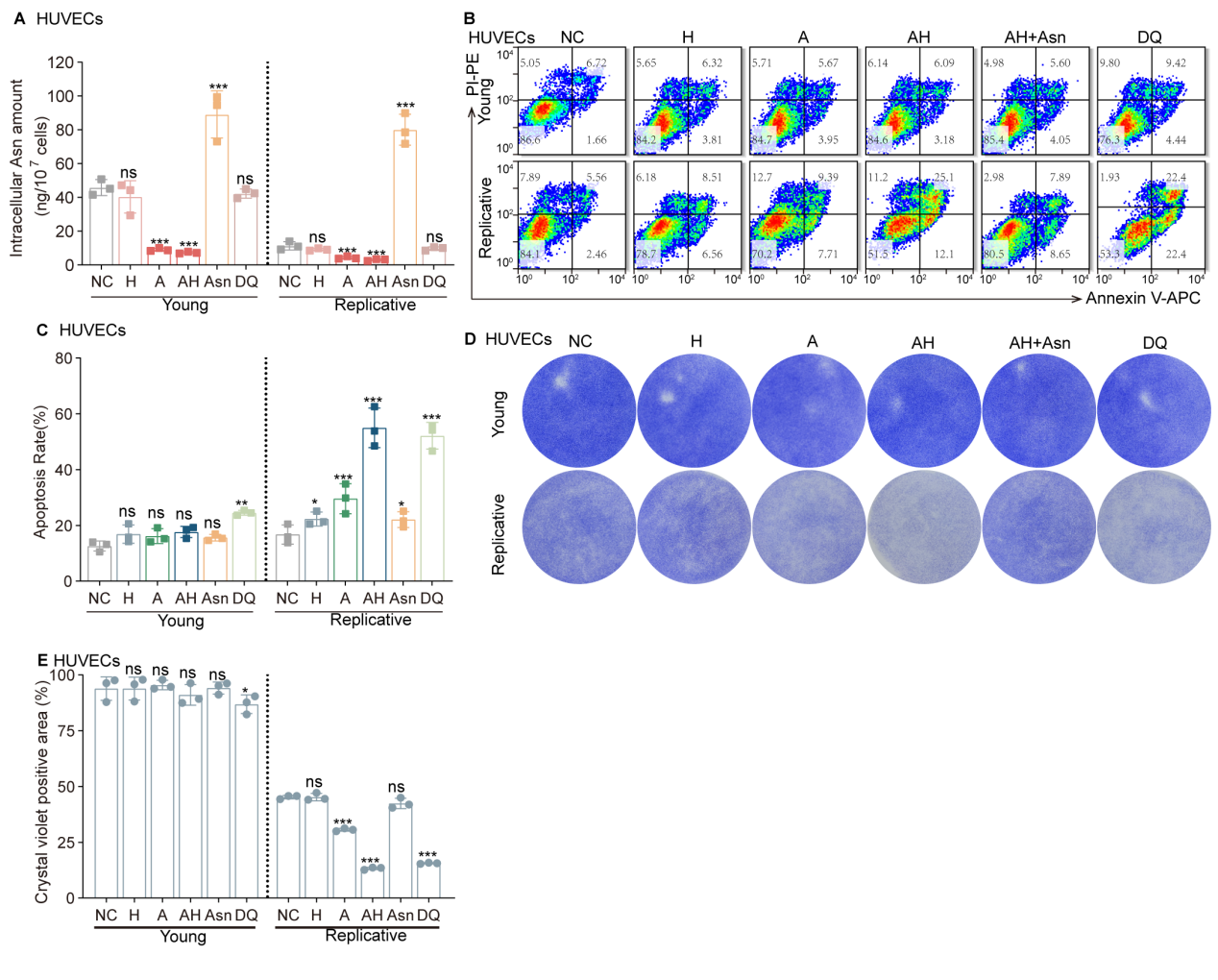


**Fig.10 AH kills senescent HUVECs.**A-E. Young and senescent HUVEC cells were subjected to the following treatments: "NC" indicates the negative control, "H" indicates cells treated with HCQ (25 μM, 3 days), "A" indicates treatment with ASNase (0.5 U/mL, 3 days), "AH" indicates simultaneous treatment with both ASNase and HCQ, "AH+Asn" or "Asn" indicates treatment with Asn (200 μM) with replenishment every 12 hours after "AH" treatment, and "DQ" indicates treatment with dasatinib (0.25 μM) and quercetin (50 μM) (n = 3 per group). (A) Detection of intracellular Asn levels by LC-MS/MS, (B) Detection of cell apoptosis by flow cytometry, (C) Analysis of apoptosis rate, (D) Crystal violet staining, and (E) Analysis of crystal violet staining positive area.Data are presented as means ± SEM. one-way ANOVA test for (A,C,E), **P* < 0.05, ***P* < 0.01, ****P* < 0.001, ns = not significant.


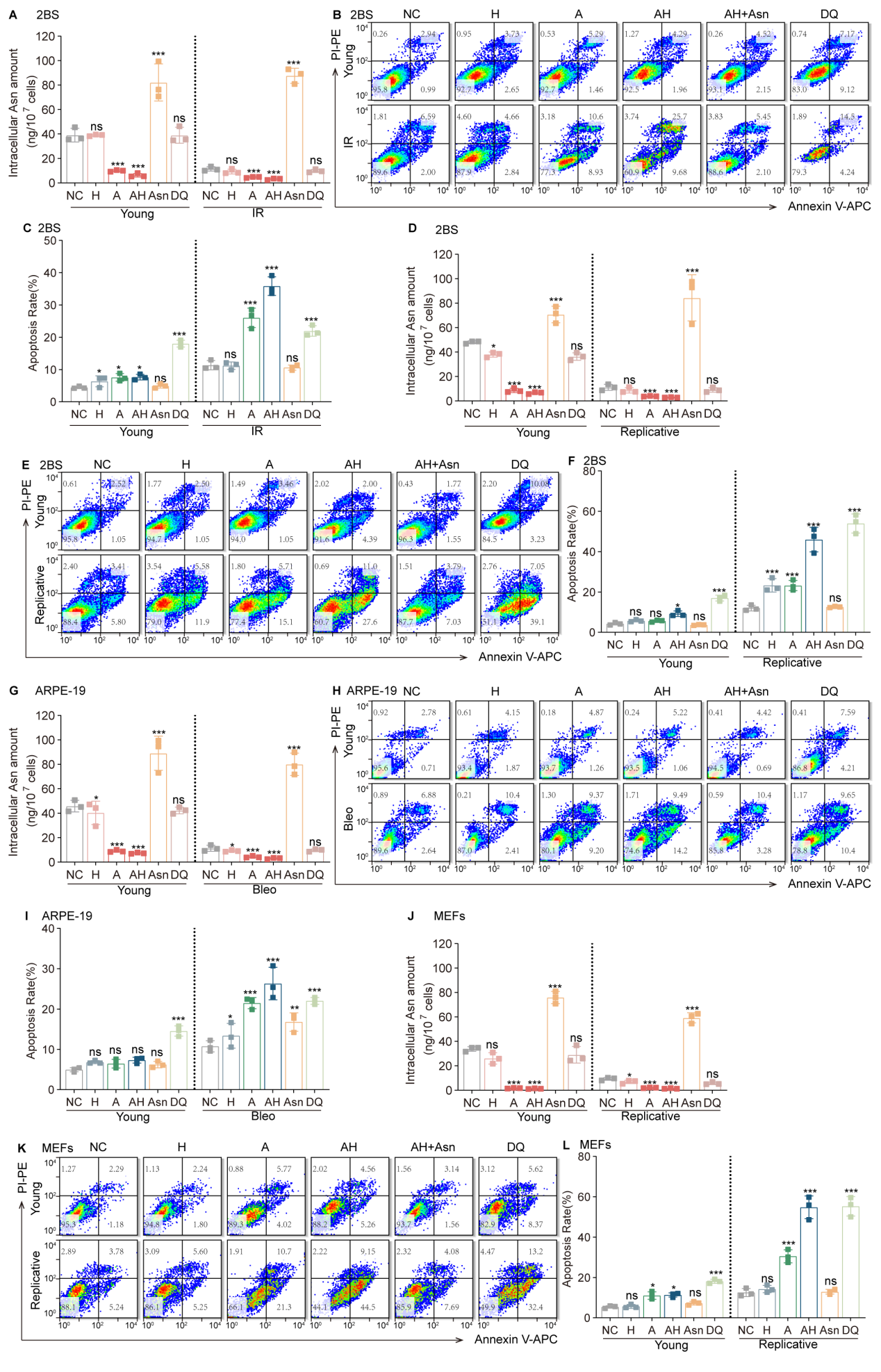


**Fig.11 AH kills various types of senescent cells.**Cells were subjected to the following treatments: "NC" indicates the negative control, "H" indicates cells treated with HCQ (25 μM, 3 days), "A" indicates treatment with ASNase (0.5 U/mL, 3 days), "AH" indicates simultaneous treatment with both ASNase and HCQ, "AH+Asn" or "Asn" indicates treatment with Asn (200 μM) with replenishment every 12 hours after "AH" treatment, and "DQ" indicates treatment with dasatinib (0.25 μM) and quercetin (50 μM) (n = 3 per group).A-C.Young 2BS cells and IR-induced senescent 2BS cells. (A) Detection of intracellular Asn levels by LC-MS/MS ,(B) Detection of cell apoptosis by flow cytometry and (C) analysis of apoptosis rate. D-F.Young 2BS cells and replicative senescent 2BS cells. (D) Detection of intracellular Asn levels by LC-MS/MS ,(E) Detection of cell apoptosis by flow cytometry and (F) analysis of apoptosis rate. G-I.Young ARPE-19 cells and bleomycin-induced senescent ARPE-19 cells. (G) Detection of intracellular Asn levels by LC-MS/MS ,(H) Detection of cell apoptosis by flow cytometry and (I) analysis of apoptosis rate.J-L.Young MEF cells and replicative senescent MEF cells.(J)Detection of intracellular Asn levels by LC-MS/MS ,(K) Detection of cell apoptosis by flow cytometry and (L) analysis of apoptosis rate.Data are presented as means ± SEM. one-way ANOVA test for (A,C-D,F-G,I-J,L), **P* < 0.05, ***P* < 0.01, ****P* < 0.001, ns = not significant.


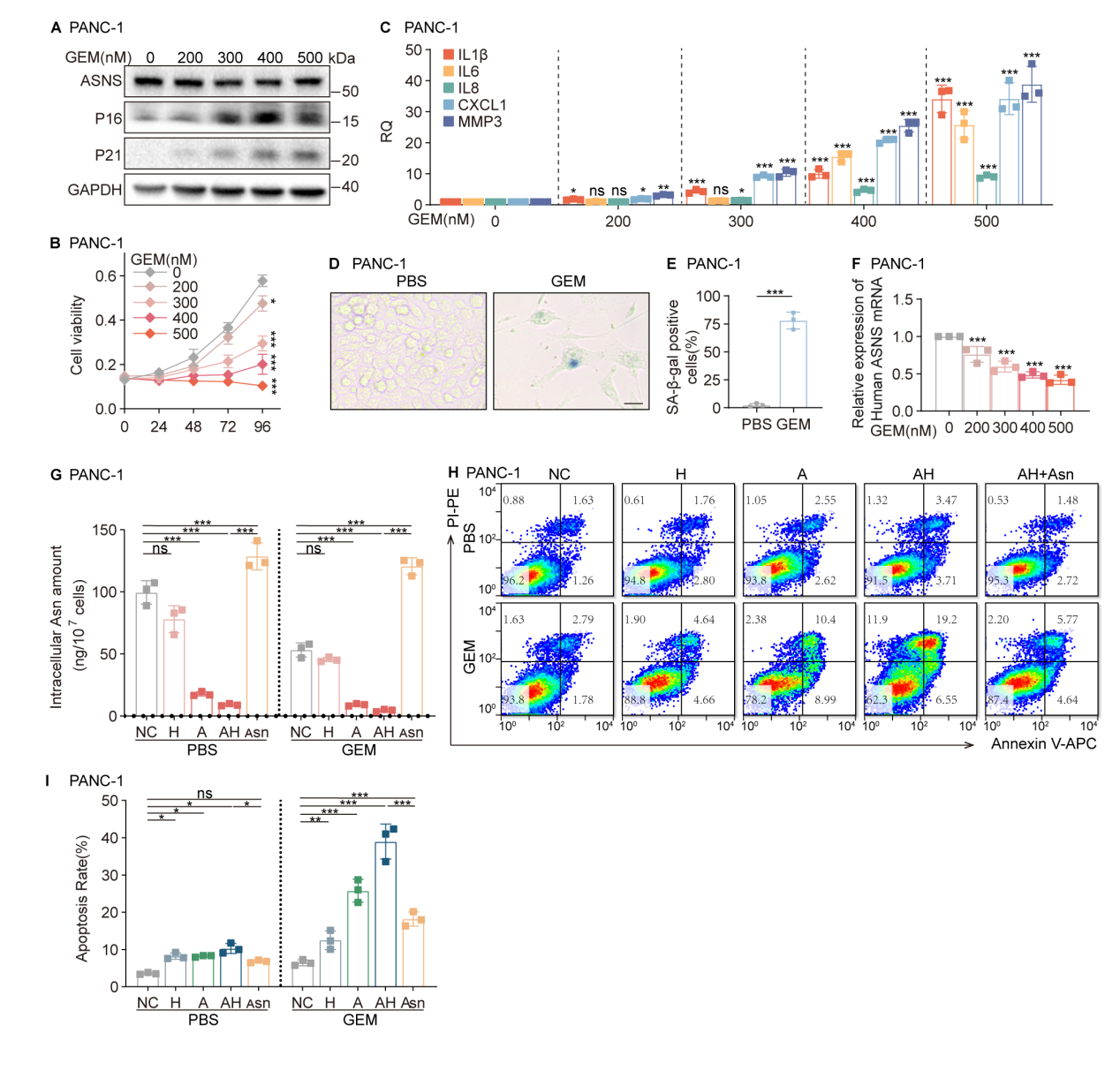


**Fig.12 AH eliminates TIS tumor cells by limiting asparagine availability.** A. Detection of ASNS and senescence-associated protein in pancreatic cancer cells PANC1 treated with gemcitabine (0-500 nM, 5 days). B. Quantification of viability of PANC1 cells (n = 3). C. Detection of SASP mRNA in PANC1 cells.D. Representative images and E. quantification of SA-β-gal staining of PANC1 cells treated with gemcitabine (500 nM, 5 days) (n = 3). Scale bar, 50 μm. F. Detection of ASNS mRNA in PANC1 cells treated with gemcitabine (0-500 nM, 5 days). G-I. PANC1 cells treated with gemcitabine (500 nM, 5 days) were subjected to the following treatments: "NC" indicates the negative control, "H" indicates cells treated with HCQ (25 μM, 3 days), "A" indicates treatment with ASNase (0.5 U/mL, 3 days), "AH" indicates simultaneous treatment with both ASNase and HCQ, "AH+Asn" or "Asn" indicates treatment with Asn (200 μM) with replenishment every 12 hours after "AH" treatment (n = 3 per group). (G) Detection of intracellular Asn levels by LC-MS/MS. (H) Detection of cell apoptosis by flow cytometry and (I) analysis of apoptosis rate. Data are presented as means ± SEM. Two-way ANOVA test for (B), unpaired two-tailed *t*-test for (E), one-way ANOVA test for (C,F,G,I),**P* < 0.05, ***P* < 0.01, ****P* < 0.001, ns = not significant.

**
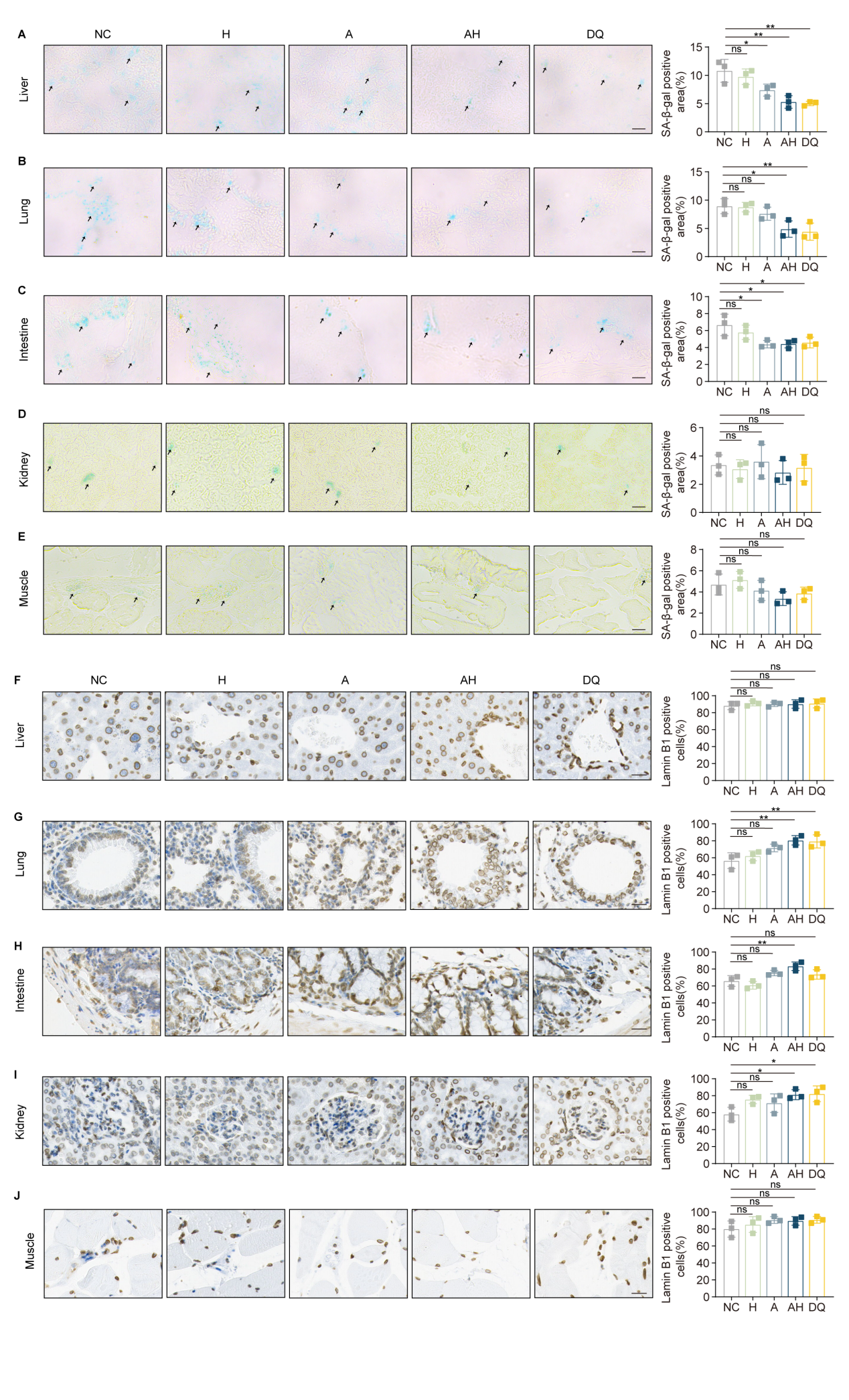
**

**Fig.13 AH deletes senescent cells of aged mice.** Eighteen-month-old mice were divided into the following groups and treated every two weeks for three consecutive days over a period of 12 weeks. NC group (n = 7): intraperitoneal injection of vehicle (saline) and oral gavage of vehicle (4% DMSO + 10% PEG400 + 86% ddH2O). HCQ group (n = 6): intraperitoneal injection of vehicle and oral gavage of HCQ(40 μg/g). ASNase group (n = 6): intraperitoneal injection of ASNase(2 U/g) and oral gavage of vehicle. AH group (n = 6): intraperitoneal injection of ASNase(2 U/g) and oral gavage of HCQ(40 μg/g). DQ group (n = 6): oral gavage of dasatinib(5 μg/g) and quercetin(50 μg/g). A-E. Representative images (left) and quantification (right) of SA-β-gal staining . (A) Liver, (B) Lung, (C) Intestine, (D)Kidney, (E) Muscle. Scale bars, 50 µm. F-J. Representative images (left) and quantification (right) of LaminB1-positive cells. (F) Liver, (G) Lung, (H) Intestine, (I)Kidney, (J) Muscle. Scale bars, 50 µm..Data are presented as means ± SEM. One-way ANOVA test for (A-J),**P* < 0.05, ***P* < 0.01, ****P* < 0.001, ns = not significant.


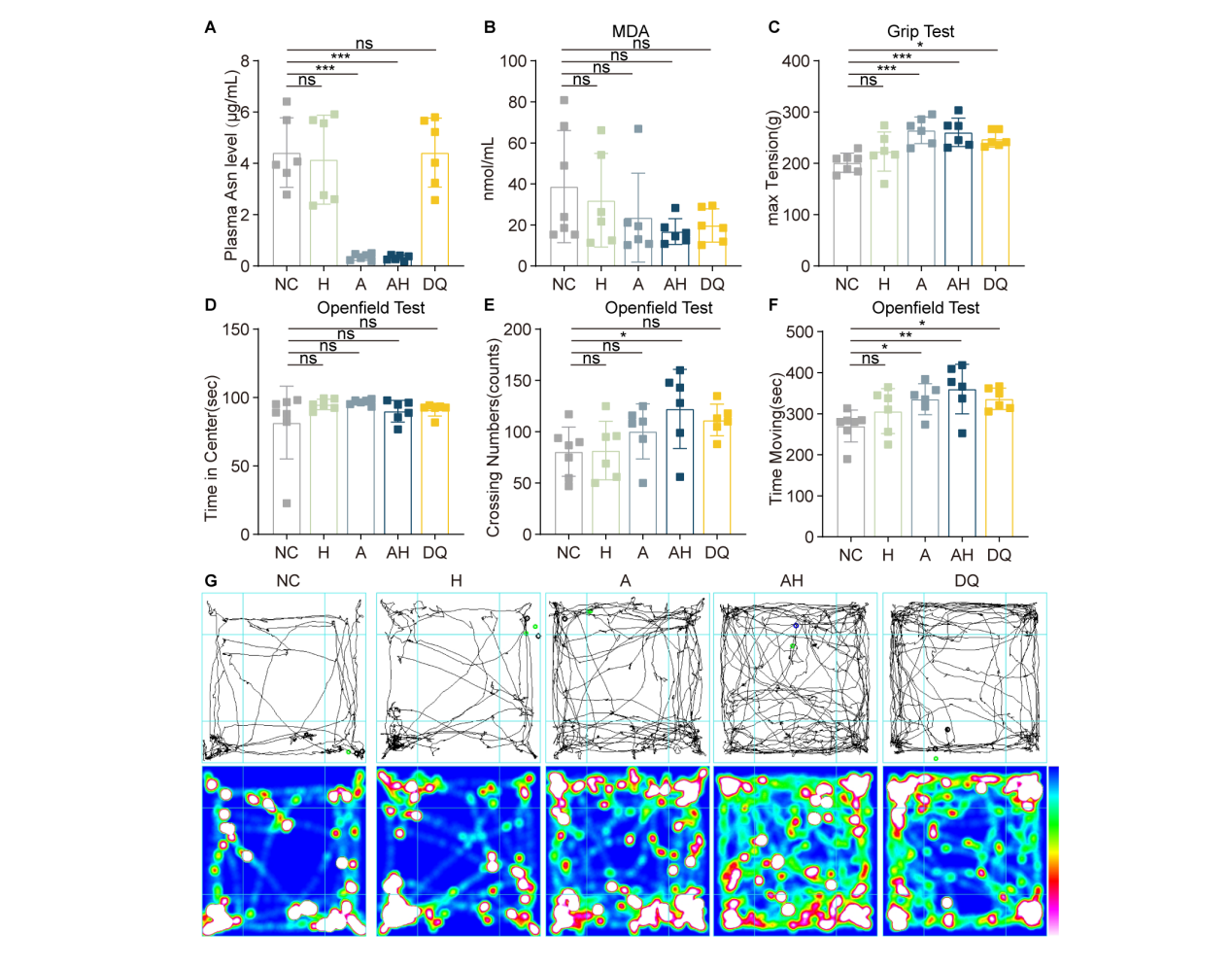


**Fig.14 AH deletes senescent cells and improves physical function of aged mice.** Eighteen-month-old mice were divided into the following groups and treated every two weeks for three consecutive days over a period of 12 weeks. NC group (n = 7): intraperitoneal injection of vehicle (saline) and oral gavage of vehicle (4% DMSO + 10% PEG400 + 86% ddH2O). HCQ group (n = 6): intraperitoneal injection of vehicle and oral gavage of HCQ(40 μg/g). ASNase group (n = 6): intraperitoneal injection of ASNase(2 U/g) and oral gavage of vehicle. AH group (n = 6): intraperitoneal injection of ASNase(2 U/g) and oral gavage of HCQ(40 μg/g). DQ group (n = 6): oral gavage of dasatinib(5 μg/g) and quercetin(50 μg/g). A. Detection of Asn levels in serum by LC-MS/MS 3 hours after treatment. B. Detection of MDA in serum. C. Maximal grip strength of four limbs of aged mice. D. Time spent in the center zone, E. number of zone crossings, and F. time spent moving of aged mice in openfield test. G. Movement trajectories and heat maps of aged mice in openfield test.Data are presented as means ± SEM. One-way ANOVA test for (A-F),**P* < 0.05, ***P* < 0.01, ****P* < 0.001, ns = not significant.


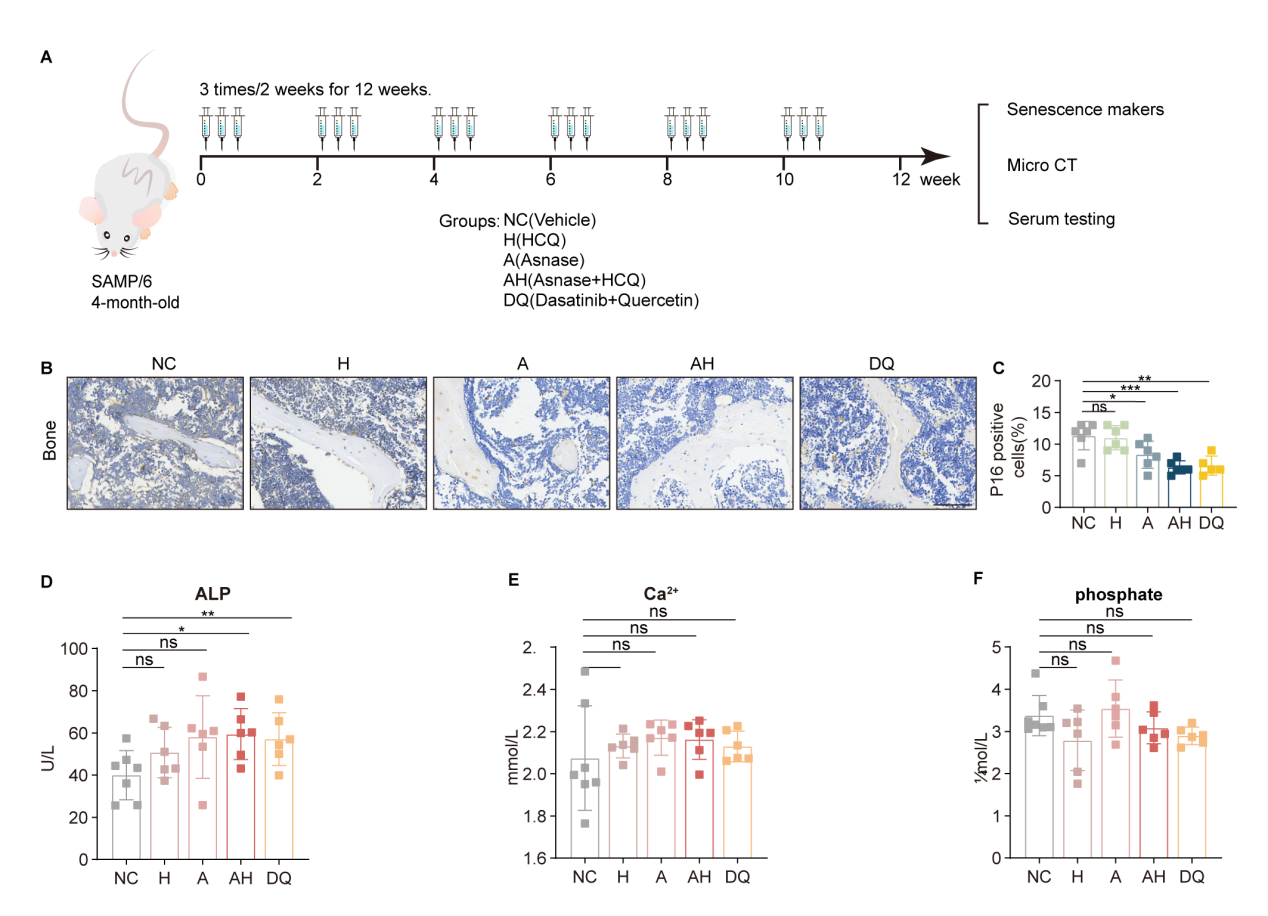


**Fig.15 AH delays the progression of senile osteoporosis.** A-F. Four-month-old SAMP/6 mice were divided into the following groups and treated every two weeks for three consecutive days over a period of 12 weeks. NC group (n = 6): intraperitoneal injection of vehicle (saline) and oral gavage of vehicle (4% DMSO + 10% PEG400 + 86% ddH2O). HCQ group (n = 6): intraperitoneal injection of vehicle and oral gavage of HCQ(40 μg/g). ASNase group (n = 6): intraperitoneal injection of ASNase(2 U/g) and oral gavage of vehicle. AH group (n = 6): intraperitoneal injection of ASNase(2 U/g) and oral gavage of HCQ(40 μg/g). DQ group (n = 6): oral gavage of dasatinib(5 μg/g) and quercetin(50 μg/g). (A) Experimental design for AH treatment of aged mice,(B)representative images and (C)quantification of P16-positive cells by IHC,(D) detection of alkaline phosphatase (ALP) levels in serum, (E) detection of calcium ion (Ca^2+^) levels in serum, (F) detection of phosphate levels in serum.Data are presented as means ± SEM. One-way ANOVA test for (B-F),**P* < 0.05, ***P* < 0.01, ****P* < 0.001, ns = not significant.


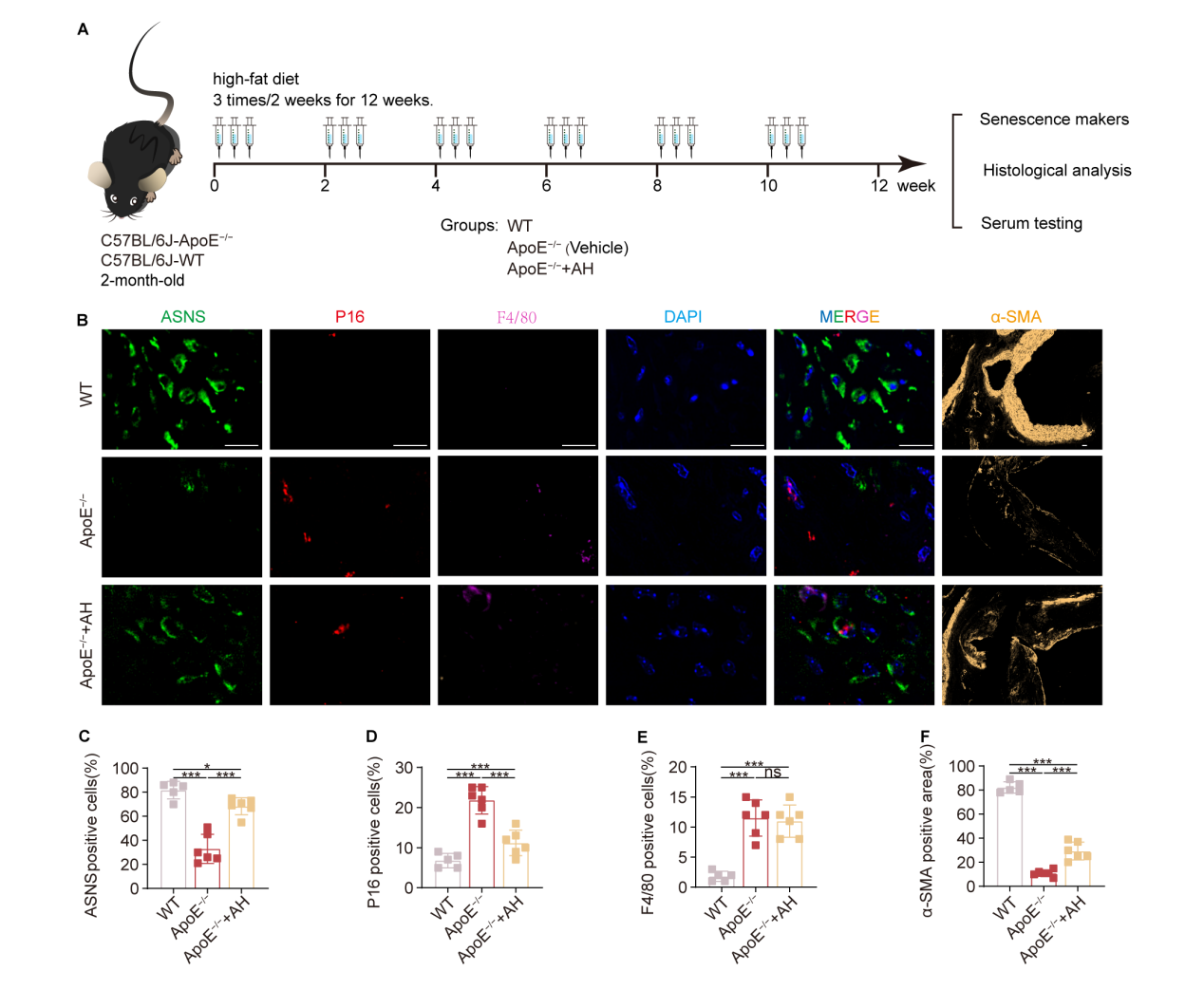


**Fig.16 AH delays the progression of atherosclerosis.** A-F. Two-month-old C57BL/6J-ApoE^−/−^ and C57BL/6J wild-type mice were divided into the following groups and treated every two weeks for three consecutive days over a period of 12 weeks. WT group: C57BL/6J wild-type mice (n = 6). ApoE^−/−^ group (n = 6): C57BL/6J-ApoE^−/−^ mice fed a high-fat diet, intraperitoneal injection of vehicle (saline), and oral gavage of vehicle (4% DMSO + 10% PEG400 +86% ddH2O). ApoE^−/−^+AH group (n = 6): C57BL/6J-ApoE^−/−^ mice fed a high-fat diet, intraperitoneal injection of ASNase(2 U/g), and oral gavage of HCQ(40 μg/g). (A) Experimental design for AH treatment of aged mice. (B) Multicolor immunohistochemistry detection of protein expression in the aortic root of mice, quantification of (C) ASNS protein expression, (D) P16 protein expression, (E) F4/80 protein expression, and (F)α-SMA protein expression in the aortic root of mice. Scale bar, 50 μm.Data are presented as means ± SEM. One-way ANOVA test for (C-F),**P* < 0.05, ****P* < 0.001, ns = not significant.


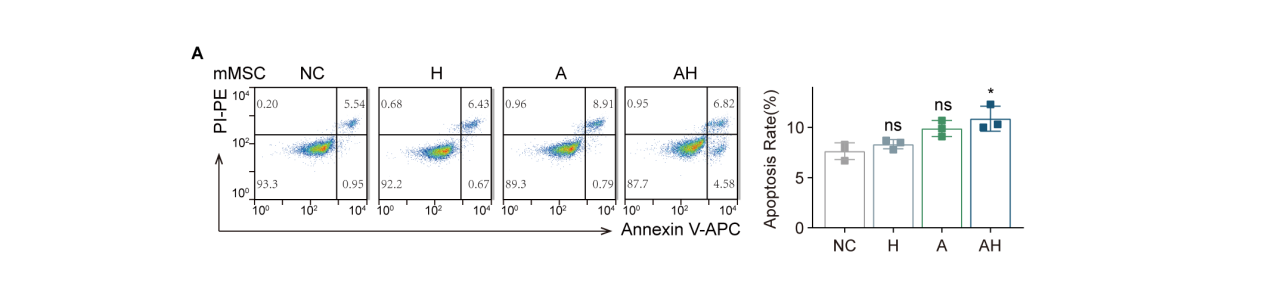


**Fig.17 AH treatment on murine mesenchymal stem cells.**murine mesenchymal stem cells were subjected to the following treatments: "NC" indicates the negative control, "H" indicates cells treated with HCQ (25 μM, 3 days), "A" indicates treatment with ASNase (0.5 U/mL, 3 days), "AH" indicates simultaneous treatment with both ASNase and HCQ(n = 3 per group).A.Detection of cell apoptosis by flow cytometry and analysis of apoptosis rate.Data are presented as means ± SEM. one-way ANOVA test for (A), **P* < 0.05, ns = not significant.


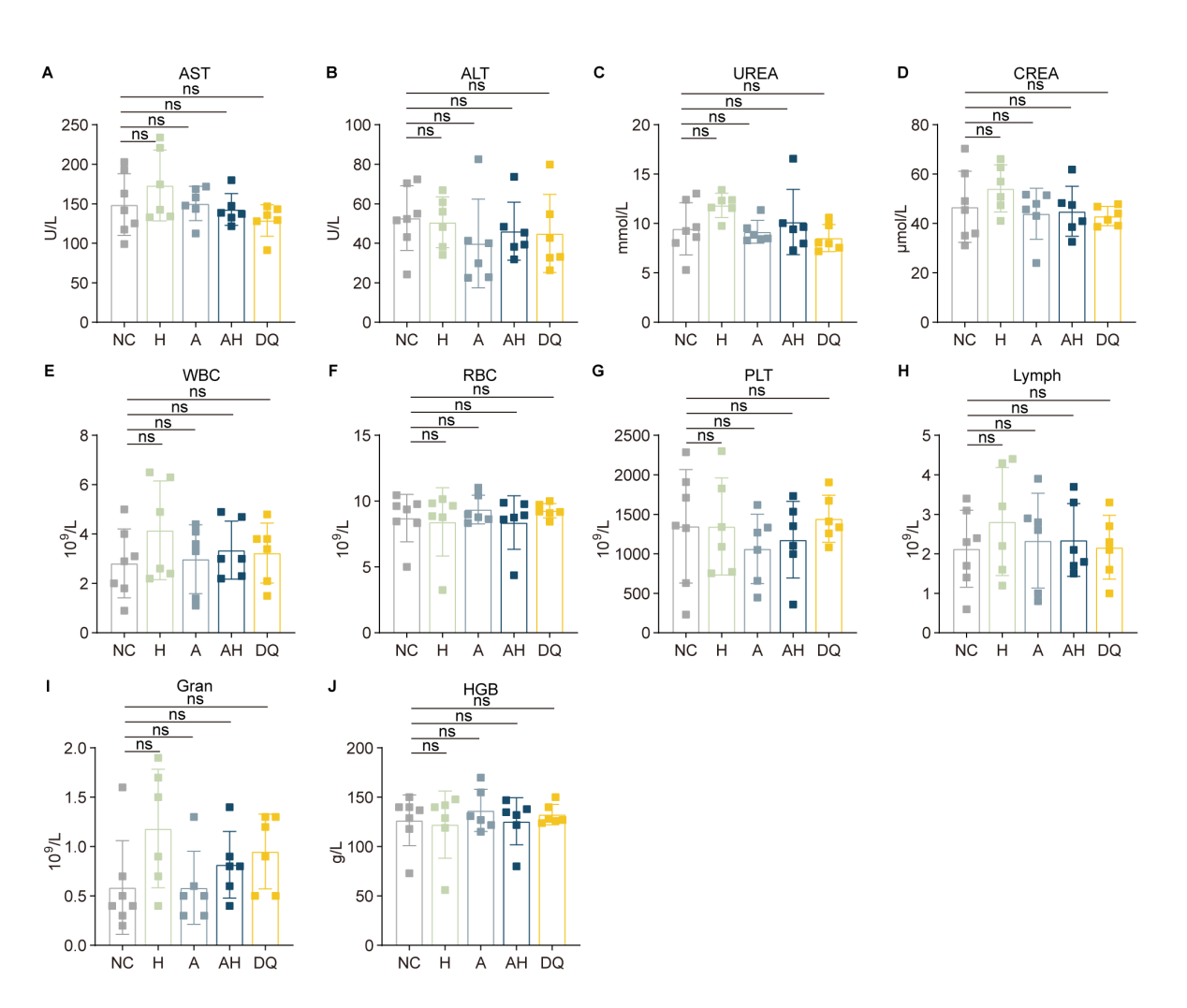


**Fig.18 The treatment of AH showed no obvious systemic toxicity.** Four-month-old SAMP/6 mice were divided into the following groups and treated every two weeks for three consecutive days over a period of 12 weeks. NC group (n = 7): intraperitoneal injection of vehicle (saline) and oral gavage of vehicle (4% DMSO + 10% PEG400 + 86% ddH2O). HCQ group (n = 6): intraperitoneal injection of vehicle and oral gavage of HCQ(40 μg/g). ASNase group (n = 6): intraperitoneal injection of ASNase(2 U/g) and oral gavage of vehicle. AH group (n = 6): intraperitoneal injection of ASNase(2 U/g) and oral gavage of HCQ(40 μg/g). DQ group (n = 6): oral gavage of dasatinib(5 μg/g) and quercetin(50 μg/g).A-D. Serum biochemical test. The levels of (A) aspartate transaminase (AST), (B) alanine transaminase (ALT), (C) carbamide (UREA), and (D) creatinine (CREA). E-J. Routine analysis of blood. The numbers of (E) white blood cells (WBC), (F) red blood cells (RBC), (G) platelets (PLT), (H) lymphocytes (Lymph), (I) granulocytes (Gran), and (J) hemoglobin (HGB).Data are presented as means ± SEM. One-way ANOVA test for (A-J),**P* < 0.05, ***P* < 0.01, ****P* < 0.001, ns = not significant.


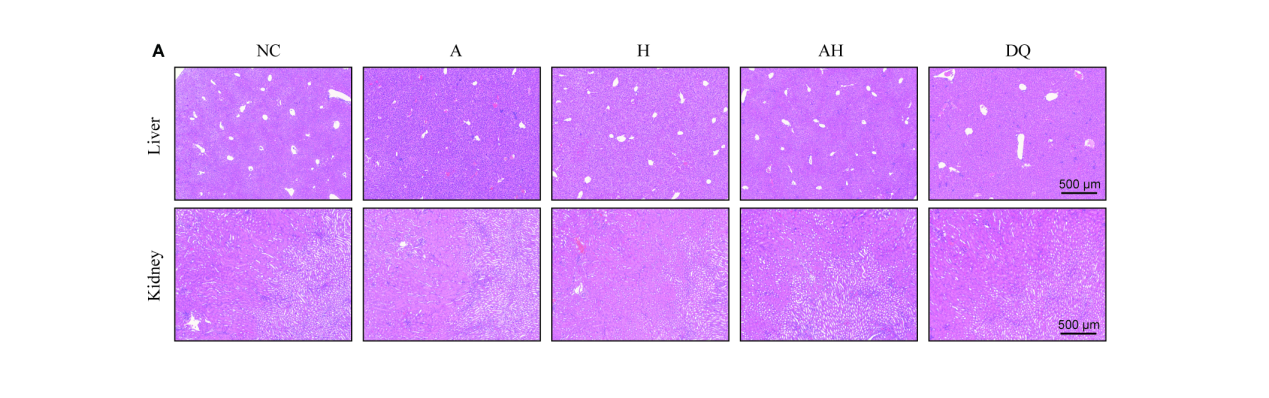


**Fig.19 Preservation of hepato-renal morphology following AH administration.** Four-month-old SAMP/6 mice were divided into the following groups and treated every two weeks for three consecutive days over a period of 12 weeks. NC group (n = 7): intraperitoneal injection of vehicle (saline) and oral gavage of vehicle (4% DMSO + 10% PEG400 + 86% ddH2O). HCQ group (n = 6): intraperitoneal injection of vehicle and oral gavage of HCQ(40 μg/g). ASNase group (n = 6): intraperitoneal injection of ASNase(2 U/g) and oral gavage of vehicle. AH group (n = 6): intraperitoneal injection of ASNase(2 U/g) and oral gavage of HCQ(40 μg/g). DQ group (n = 6): oral gavage of dasatinib(5 μg/g) and quercetin(50 μg/g).A. Representative H&E-stained sections of hepatic and renal tissues of mice.


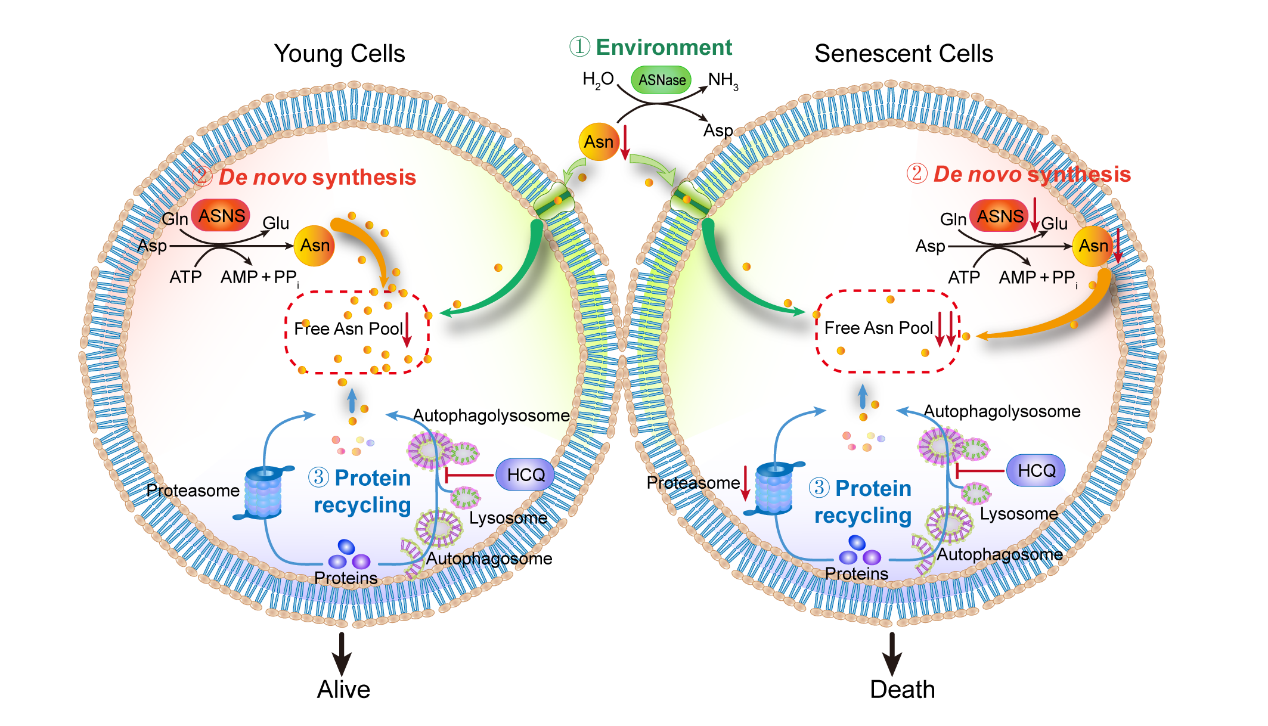


**Fig.20 A model of cellular asparagine source and AH treatment kills senescent cells effectively and selectively.** Cells can acquire free Asn through three mechanisms: ①extracellular uptake; ②*de novo* synthesis via ASNS; and ③protein recycling. In senescent cells, there is a defect in ASNS. Treatment with ASNase to deplete extracellular Asn, combined with HCQ to inhibit autophagy-derived Asn, results in a severe depletion of Asn and subsequent senescent cell death. Conversely, young cells, which express ASNS normally, are not affected by ASNase and HCQ treatments.
